# Mu-driven transposition of recombinant mini-Mu unit DNA in the *Corynebacterium glutamicum* chromosome

**DOI:** 10.1007/s00253-018-8767-1

**Published:** 2018-02-01

**Authors:** Natalya V. Gorshkova, Juliya S. Lobanova, Irina L. Tokmakova, Sergey V. Smirnov, Valerii Z. Akhverdyan, Alexander A. Krylov, Sergey V. Mashko

**Affiliations:** Ajinomoto-Genetika Research Institute, 1-st Dorozhny proezd, 1-1, Moscow, Russian Federation 117545

**Keywords:** Cre-mediated excision, Excisable enhancer, Fluorescence proteins, Intrachromosomal amplification, Random integration, Replicative transposition

## Abstract

A dual-component Mu-transposition system was modified for the integration/amplification of genes in *Corynebacterium*. The system consists of two types of plasmids: (i) a non-replicative integrative plasmid that contains the transposing mini-Mu(**LR**) unit bracketed by the **L**/**R** Mu ends or the mini-Mu(**LER**) unit, which additionally contains the enhancer element, **E**, and (ii) an integration helper plasmid that expresses the transposition factor genes for MuA and MuB. Efficient transposition in the *C. glutamicum* chromosome (≈ 2 × 10^−4^ per cell) occurred mainly through the replicative pathway via cointegrate formation followed by possible resolution. Optimizing the **E** location in the mini-Mu unit significantly increased the efficiency of Mu-driven intramolecular transposition–amplification in *C. glutamicum* as well as in gram-negative bacteria. The new *C. glutamicum* genome modification strategy that was developed allows the consequent independent integration/amplification/fixation of target genes at high copy numbers. After integration/amplification of the first mini-Mu(**LER**) unit in the *C. glutamicum* chromosome, the **E**-element, which is bracketed by *lox*-like sites, is excised by Cre-mediated fashion, thereby fixing the truncated mini-Mu(**LR**) unit in its position for the subsequent integration/amplification of new mini-Mu(**LER**) units. This strategy was demonstrated using the genes for the citrine and green fluorescent proteins, *yECitrine* and *yEGFP*, respectively.

**Electronic supplementary material:**

The online version of this article (10.1007/s00253-018-8767-1) contains supplementary material, which is available to authorized users.

## Introduction

Since its discovery in 1957, as an l-glutamate-producing non-pathogenic Gram-positive soil bacterium from the *Actinomyces* branch and its classification as a “generally recognized as safe” (GRAS) organism, *Corynebacterium glutamicum* has become a workhorse for the large-scale industrial production of amino acids, chemicals, materials, fuels, and various proteins (Becker and Wittmann 2012). Recent progress in the characterization and targeted engineering of the metabolism of *C. glutamicum* is mainly based on high-throughput omics techniques such as genomics (Ikeda and Nakagawa 2003; Kalinowski et al. 2003), transcriptomics (Glanemann et al. 2003; Inui et al. 2007; Wendisch 2003), proteomics (Hermann et al. 2001; Li et al. 2007), metabolomics (Bartek et al. 2010; Woo et al. 2010), and fluxomics (Kjeldsen and Nielsen 2009; Marx et al. 1996; Shinfuku et al. 2009; Wittmann and Heinzle 2002), as well as the accelerated development of highly efficient genetic tools for this organism.

Currently, there are many approaches for chromosomal editing of *C. glutamicum*. Most of these techniques are based on the application of various types of plasmids (Nešvera and Pátek 2008; Tauch 2005), which allow the deletion, substitution, and overexpression of target genes (Kirchner and Tauch 2003; Nešvera and Pátek 2011). Unfortunately, to our knowledge, a perfect analogue of the λRed/RecET-based recombineering approach for the high-efficiency integration of double-stranded PCR products with rather short homologous arms into targeted loci of the bacterial chromosome (reviewed in (Court et al. 2002))—a method developed for *Escherichia coli* and several other gram-negative bacteria (Datsenko and Wanner 2000; Katashkina et al. 2009; Swingle et al. 2010)—has been recently described for *C. glutamicum* only in one publication (Huang et al. 2017), though the corresponding experiments have been earlier announced (Ma et al. 2015). Moreover, the already published RecT-dependent (Binder et al. 2013; Cho et al. 2017; Jiang et al. 2017) or annealing protein-independent (Krylov et al. 2014) recombination approaches between short single-stranded oligonucleotides and a targeted locus in the *C. glutamicum* chromosome are good starting points. Additionally, integrative plasmid vectors have also been constructed based on various corynephages, and these carry DNA elements that enable phage-governed site-specific recombinant DNA integration (Moreau et al. 1999; Oram et al. 2007).

In addition, several different mini-transposons that work according to the so-called cut-and-paste mechanism of transposition (miniTn*31831*, Tn*5*-based; Tn1*3655*) have been successfully used for the integration of recombinant DNA at random locations in the *C. glutamicum* chromosome (Suzuki et al. 2006; Tsuge et al. 2007).

However, the integration and possible amplification of target genes in the chromosome of *Escherichia coli* and closely related gram-negative bacteria is known to be efficiently achieved using a system based on phage Mu-driven transposition that was initially characterized and practically exploited more than 30 years ago (Castilho et al. 1984; Chaconas et al. 1981a, b).

The Mu phage undergoes two alternative transposition pathways at different stages of its life cycle that differ in their donor substrate configuration and fate of the transposition products: (i) nick-join-reparative transposition, which results in the integration of linear Mu DNA bracketed by specific Mu **L** and **R** ends, into random sites spaced 5 bp apart in the bacterial chromosome during Mu phage infection; and (ii) nick-join-replicative transposition, which occurs through the formation of a cointegrate structure that is obligatory for replication during phage lytic growth (Au et al. 2006; Choi et al. 2014; Harshey 2012, 2014). The Mu-driven replicative transposition pathway provided by an artificial dual-component system was previously extensively used for genome editing of gram-negative bacteria (Akhverdyan et al. 2011). In this system, the first component is an “integrative” plasmid that contains transposing DNA in the form of either a mini-Mu(**LR**) unit bracketed by **L** and **R** ends or a mini-Mu(**LER**) unit in which an enhancer element, **E**, is properly arranged between **L/R** to positively influence the efficiency of transposition (Leung et al. 1989). The second component is an integration helper plasmid that contains inducible genes for the MuA and MuB transposition factors, thus enabling integration of the mini-Mu unit located in the first plasmid. Supplied *in trans* on an unlinked/non-transposed compatible helper plasmid, these Mu*AB* genes can be eliminated from recipient cells after mini-Mu unit transposition into the bacterial genome (Akhverdyan et al. 2011). Among the alternative systems typically used for the Mu-driven integration of recombinant DNA into a heterologous host genome, electroporation of an in vitro-assembled linear mini-Mu unit in combination with the MuA transposase has successfully resulted in Mu-driven reparative transposition into the chromosome of different organisms, including not only gram-negative bacteria (Lamberg et al. 2002; Lanckriet et al. 2009) but also gram-positive bacterial species (Pajunen et al. 2005) and yeasts as well as mouse and human genomes (Paatero et al. 2008).

In the present study, an expressed dual-component Mu-driven system efficiently transposed target genes in *Corynebacterium glutamicum* mainly through the nick-join-replicative pathway. Moreover, this work confirmed that proper placement of the **E** element in the mini-Mu unit structure could increase the efficiency of Mu-driven integration, especially the efficiency of intrachromosomal amplification, in the *C. glutamicum* chromosome as well as in gram-negative strains (Akhverdyan et al. 2011). Using specially constructed Cre-excisable cassettes with an **E** element bracketed by *lox*-like sites as part of a mini-Mu(**LER**) unit, a new genome modification strategy was developed and adjusted, providing a new tool for the *C. glutamicum* genetic toolbox. This strategy consists of several repeated stages in which the *i*th stage includes three consecutive steps: (i) selective MuAB-dependent integration of the mini-Mu(**LER**)-*i* unit into a random location on the bacterial genome, followed by (ii) its Mu-driven intrachromosomal amplification and (iii) fixation of truncated mini-Mu(**LR**)-*i* units at their new positions, due to Cre-mediated excision of their **E** elements and selective markers artificially bracketed by *lox*-like elements. An analogous three-step genome modification strategy could be performed with a mini-Mu(**LER**)-(*i* + 1) unit, beginning with its MuAB-driven integration and amplification, provided that all previously transposed mini-Mu-*k* units (where *k =* 1, 2, …, *i*) lost the **E** element from their structures and are stably maintained. The application of this novel strategy was successfully demonstrated in *C. glutamicum*.

## Materials and methods

### Strains, plasmids, and growth conditions

All of the strains and plasmids used in this study are described in Table 1. The *Corynebacterium glutamicum* strains were grown at 32 °С on Brain Heart Infusion (BHI) medium (Difco, USA). When needed, the corresponding antibiotics were added at the following final concentrations: 1 μg/mL of gentamicin (Gm), 25 μg/mL of kanamycin (Km), 1 μg/mL of tetracycline (Tc), and 250 μg/mL (normal) or 750 μg/mL (high) of streptomycin (Sm).

**Table 1:** Strains and plasmid used in the present study

Strain and plasmid	Relevant characteristics	Reference or source
*C. glutamicum* strains		
ATCC13869	Wild type	Laboratory collection
ATCC13032	Biotin-auxotrophic wild type	VKPM B-41
ATCC13869 recA^−^	ATCC13869 with deletion *recA* gene	Laboratory collection
MB001	ATCC13032 with in-frame deletion of prophages CGP1, CGP2, CGP3	Baumgart et al. 2013
1YK	ATCC13869 with integration of mini-Mu(**LER**)-YK in the chromosome	This work
2YK	1YK with the second amplified copy of mini-Mu(**LER**)-YK in the chromosome	This work
3YK	2YK with the third amplified copy of mini-Mu(**LER**)-YK in the chromosome	This work
1Y	Derivative of the 1YK strain obtained due to Cre-mediated excision a DNA fragment bracketed by *lox66*/*lox71* sites and consisted of (Km^R^, Sm^R^) and **E** element, from the single copy of the mini-Mu unit	This work
2Y	Derivative of the 2YK strain consisted of two copies of the mini-Mu unit truncated by Cre-mediated excision of DNA fragment bracketed by *lox66*/*lox71* sites	This work
3Y	Derivative of the 3YK strain consisted of three copies of the truncated mini-Mu units	This work
*E. coli* strains		
TG1	F^−^ Δ(*lac-pro*) *supE thi hsd*Δ5 [F′ *traD*36 *proAB*^*+*^ *lacI*^q^ *lacZ*ΔM15]	VKM IMG-341
BW25141	*lacI* ^q^ *rrnB*_T14 ∆_ *lacZ*_*WJ16*_ *∆phoBR580 hsdR514 ∆araBAD*_AH33_	Datsenko and Wanner 2000
	*∆rhaBAD*_LD78_ *galU95 endA*_BT333_ *uidA(∆MluI)::pir* ^*+*^ *recA1*	
Plasmids		
pTP310	Tc^R^; pRK310 with 5.7 kb *BamH*I fragment from pUC-MuAB, containing Mu*AB*, *ner*, and *c*^ts^ genes	Abalakina et al. 2008
pBGR10	Gm^R^, Km^R^; derivative of pBHR1, contains *aac1* gene of pBGEA10	Ishikawa et al. 2008
pVK9	Km^R^; shuttle vector: *C. glu* pCG1 replicon (Ozaki et al. 1984); *E. coli* ColE1replicon (Backman et al. 1979)	Nakamura et al. 2006
pVK-Gm^R^	pVK9 derivative with Gm^R^ marker	This work
pVK9-Gm^R^-(*lacI*^Q^-P_*tac*_-Mu*AB*)	pVK-Gm^R^ derivative with cloned *lacI*^Q^-P_*tac*_-Mu*AB*	This work GenBank KP272129
pAH162	Тс^R^; derivative of R6K with *oriRγ*	Haldimann and Wanner 2001; Posfai et al. 1994
pMIV5-[FRT-KmR-FRT]-SmR-Mob	Ap^R^; Km^R^; Sm^R^; pMIV5-Mob[FRT-Km^R^-FRT] with the 1.9-kb *EcoR*V fragment containing the *strAB* genes under the control of *M. methylotrophus* P17 promoter	Abalakina et al. 2008
pAH-mini-Mu(**LR**)-K	Tc^R^; Sm^R^; Km^R^; pAH162-Mu*att***L**-T_*his*_*-P*_17*Mme*_ [*lox66*- *strAB*-Km^R^-*lox71*]-T_*deo-*_Mu*att***R**	This work
pKT139	Plasmid pFA6a–link–yECitrine–*SpHIS5*, coding yECitrine	Sheff and Thom 2004 EUROSCARF, accession numbers P30186
pAH-mini-Mu(**LR**)-YK	Tc^R^; Sm^R^; Km^R^; derivative of pAH-mini-Mu(**LR**)-K; contains *yECitrine* inside mini-Mu; pAH162-Mu*att***L**-T_*his*_ *-P*_17*Mme*_*yECitrine*[*lox66*- *strAB*-Km^R^-*lox71*]-T_*deo-*_Mu*att***R**	This work
pAH-mini-Mu(**LER**)-YK	Tc^R^; Sm^R^; Km^R^; derivative of pAH-mini-Mu(**LR**)-YK, contains **E**, E_direct_, inside mini-Mu; pAH162-Mu*att***L**-T_*his*_ *-P*_17*Mme*_*yECitrine*[*lox66*- *strAB*-**E**-Km^R^-*lox71*]-T_*deo-*_Mu*att***R**	This work
pAH-mini-Mu(**LER**)-YK	Tc^R^; Sm^R^; Km^R^; derivative of pAH-mini-Mu(**LR**)-YK, contains E_converse_ inside mini-Mu	This work
pKT128	pFA6a–link–yEGFP–SpHIS5 coding yEGFP	Sheff and Thorn 2004 EUROSCARF, accession numbers P30174
pAH-mini-Mu(**LER**)-GK	Tc^R^; Sm^R^; Km^R^;; derivative of pAH-mini-Mu(**LER**)-YK, in which *yECitrine* changed for *yEGFP*	This work
p06-P_*dapA*_	Cm^R^; derived of pVK9; contains *E. coli* replicon p15A, promotor P_*dapА*_	Laboratory collection
p06-Cm^R^-(P_*dapA*_-Сre)	Cm^R^; p06-P_*dapA*_, contains 1.1 kb *Sal*I-*Kpn*I fragment with *cre* gene under control of P_*dapA*_	This work
pCM110	Tc^R^; *M. extorquens* AM1 expression vector of IncP group	Marx and Lidstrom 2001
pCM110-Gm^R^	Gm^R^; derivative of pCM110 used in the present study as a wide-host-range plasmid vector of IncP group for short-gun cloning *C. glutamicum* DNA fragments in *E. coli*	This work
pMIV5	ApR; pMW119 with mini-Mu(**LR**) unit containing MCS from pUC59	Abalakina et al. 2008
pOK12	Km^R^; cloning vector	Vieira and Messing 1991
pOK17PR	Km^R^; pOK12, contains 0.1 kb *Cla*I-*Eco*RI fragment with *M. methylotrophus* promoter P17	Laboratory collection

*E. coli* strains were grown at 37 °C on Luria-Bertani (LB) medium (Sambrook and Russell 2001). When needed, the corresponding antibiotics were added at the following final concentration: 200 μg/mL of ampicillin (Ap), 20 μg/mL of Tc, 50 μg/mL of Sm, 10 μg/mL of Gm, and 40 μg/mL of Km.

### Recombinant DNA experiments

All of the oligonucleotides used in this study are described in Table S1. The restriction, ligation, electrophoresis, and Ca^2+^-dependent transformation of *E. coli* cells were performed according to standard protocols (Sambrook and Russell 2001). Plasmid and genomic DNA were isolated using a QIAPREP spin kit (QIAGEN, Hilden, Germany) and Genomic DNA Purification Kit (Thermo Fisher Scientific, Waltham, Ma, USA), respectively. Restriction enzymes, T4 DNA ligase, Long PCR Enzyme Mix, and High Fidelity PCR Enzyme Mix were purchased from Thermo Fisher Scientific (Waltham, MA, USA) and *Taq* DNA polymerase was purchased from Sileks-M (Moscow, Russia). These enzymes were used according to the manufacturers’ instructions. DNA sequencing was performed commercially by Genotekhnologiya (Moscow, Russia). The “Supplementary Material” contains detailed methods for the construction of the integration helper plasmid, pVK-*lacI*^Q^-P_*tac*_-Mu*AB* (GenBank accession no. MG014199, Fig. S1); the excision helper plasmid, p06-P_*dapA*_-*cre* (GenBank accession no. MG014197, Fig. S2); and all of the integrative plasmids: pAH-mini-Mu(**LR**)-YK (Fig. S3), pAH-mini-Mu(**LER**)-YK (GenBank accession no. MG014198, Fig. S4), pAH-mini-Mu($\mathbf{L}\overleftarrow{\mathbf{E}}\mathbf{R}$)-YK (Fig. S4), and pAH-mini-Mu(**LER**)-GK (Fig. S5).

### Electroporation protocol for *C. glutamicum*

The protocol presented in this study is the result of electroporation (electrotransformation) optimization, which was performed to achieve the highest possible rate of *C. glutamicum* ATCC13869 cell transformation with native superhelical (SH) plasmid DNA (e.g., purified from *E. coli* cells). A 250-μL sample of an overnight culture of *C. glutamicum* was added to 5 mL of BHI liquid medium, and the cells were grown at 32 °С to an OD_595_ of 1.5–2 over approximately 1.5–2 h. Then, Ap was added (100 μg/mL), and the cells were incubated for one additional hour. Next, the cells were cooled to + 4 °C and 5 mL of cell culture was harvested by centrifugation. For electrotransformation with a MicroPulser™ (Bio-Rad, Hercules, CA, USA), the cells were washed three times in 10% glycerol at + 4 °C and concentrated to 50 μL in 10% glycerol. These electrocompetent cells were mixed with 100 ng of plasmid DNA immediately prior to transformation and transferred to a 0.1-cm sterile, cold electrode chamber for electroporation via a 2.0 kV, 25 μF и 200 Ω pulse. The cells were immediately diluted with 1 mL of BHI medium and incubated for approximately 2 h at 32 °C with shaking, followed by selection of the desirable transformants using solid selective media (1.5% agar) for 2–3 days at 32 °C. The typical transformation efficiency of the ATCC13869 strain was ≈ 1 × 10^−3^/100 ng DNA/surviving cells. To achieve high transformation efficiency, cells of the MB001 (DSM102070) and ATCC13032 strains were diluted with 1 mL of BHI medium and incubated at 46 °C for 6 min immediately after electrotransformation. This modification significantly increased the transformation efficiencies of these two latter strains, but the corresponding values still did not exceed 10^−4^/100 ng DNA/surviving cells for MB001 and 1.5 × 10^−5^/100 ng DNA/surviving cells for ATCC13032.

### Integration of the mini-Mu unit in *C. glutamicum* chromosome

The electroporation protocol described above, with slight modification, was used for the MuAB-driven integration of a mini-Mu unit into the *C. glutamicum* chromosome. Briefly, *C. glutamicum* strain of interest that already possessed the integration helper plasmid, pVK-*lacI*^Q^-P_*tac*_-Mu*AB* seeded densely (with start OD 0.4–0.8), were grown at 37 °С overnight. A 500-μL sample of an overnight culture of *C. glutamicum* was added to 5 mL of BHI liquid medium, and the cells were grown at 32 °С to an OD_595_ of 1.5–2 over approximately 1.5–2 h. Induction of the expression of the Mu*AB* genes was achieved by adding 1.5 mМ IPTG into the BHI medium during the recovery step. The targeted integrants were selected on solid BHI medium supplemented with Km. Integration was confirmed by PCR using the primer pair P37/P38. For the pAH-mini-Mu(**LER**)-YK integrative plasmid in particular, the obtained Km^R^ clones in which the replicative transposition pathway occurred through cointegrate formation were detected by their Sm^HR^ and Tc^R^ phenotypes. Conversely, the obtained Km^R^ clones in which the reparative transposition pathway (or replicative transposition followed by fast cointegrate resolution) occurred had Sm^R^ and Tc^S^ phenotypes. The absence of the helper plasmid was determined by a Gm^S^ phenotype in the obtained clones.

In one specially indicated case, preparations of covalently closed but relaxed plasmid DNA were used for integration. For relaxation, the native integrative plasmid was hydrolyzed at a unique restriction site (*Sal*I) located in the mini-Mu portion of the plasmid, followed by recircularization of the plasmid via treatment with T4 DNA ligase at a low DNA concentration (≈ 1 μg/mL).

### Intrachromosomal amplification of the mini-Mu(**LER**) unit

The resolved cointegrate carrying the mini-Mu(**LER**) unit in the chromosome and the pVK-*lacI*^Q^-P_*tac*_-Mu*AB* as a plasmid was grown overnight with aeration at 37 °C in liquid BHI medium supplemented with 1.5 mM IPTG. Then, cells were seeded in series of dilutions (from 10^−2^ to 10^−5^) onto solid BHI medium containing a high concentration of Sm. Single colonies that grew on these plates were considered Sm^HR^ cells and likely contained amplified mini-Mu units. Amplification efficiency was calculated as the number of Sm^HR^ colonies per total number of seeded Sm^R^ cells estimated by CFU. Finally, Sm^HR^ clones were cured of the integration helper plasmid by dual-reseeding and aerobic cultivation in liquid BHI medium at 37 °C for 48 h, followed by plating for the selection of Gm^S^ phenotypes.

### Excision of the *lox*-bracketed DNA fragment from the mini-Mu units

Helper plasmid p06-P_*dapA*_-*cre* was transformed into the *C. glutamicum* integrants. Clones with this plasmid were selected on solid BHI medium supplemented with Cm at 30 °C after 48 h of growth. The selected Cm^R^ clones were seeded from single colonies onto solid BHI medium without antibiotics at 30 °C. The resulting clones were tested on solid BHI medium containing Km and Cm for the presence of the Km^S^, Sm^S^, and Cm^R^ markers.

### Southern blotting analysis

Southern hybridization was performed in accordance with conventional protocols (Sambrook and Russell 2001) using the following equipment: BrightStar™-Plus Positively Charged Nylon Membrane (Thermo Fisher Scientific, Waltham, MA, USA), VacuGene XL Vacuum Blotting System (GE Healthcare, Chicago, IL, USA), and a Hybridization Oven/Shaker (former Amersham Biosciences). DNA labeling with Biotin-11-dUTP (Thermo Fisher Scientific, Waltham, MA, USA) was performed in a standard 50 μL PCR reaction with the necessary pairs of primers and templates, and 0.2 mM Biotin labeling mix and *Taq* DNA polymerase. The Biotin labeling mix consists of 2 mM dGTP, 2 mM dATP, 2 mM dCTP, 1.3 mM dTTP, and 0.7 mM Biotin-11-dUTP aqueous solution. Biotin chromogenic detection kits (Thermo Fisher Scientific, Waltham, MA, USA) were used to detect the DNA probes after Southern hybridization. The primer pairs used for the PCR amplification of the probes were P37/P38 for *kan* and P22/P23 for *yECitrine* (Table S1).

### Fluorescence intensity assay

Colonies of target cells contained the *yECitrine* and/or *yEGFP* genes; control cells without either of these genes were picked, and 200-μL cellular suspensions of these cells were prepared separately in 96-well plates (GBO, Kremsmünster, Austria). Optical density (OD_595_) and fluorescence intensity (*F*) were measured using the Safire™ plate reader (Tecan, Männedorf, Switzerland). The excitation/emission wavelengths for yECitrine and yEGFP were 522/560 nm and 490/522 nm, respectively. The fluorescence intensity of a blank sample with no cells was established as the background fluorescence (*F*_background_). The average OD_595_ of the samples was between 0.2–0.3. Relative fluorescence intensity (RF) was calculated according to the equation RF = [(*F*_target_ – *F*_background_)/OD_target_] and expressed in arbitrary units.

### Determination of the mini-Mu unit integration points

Chromosomal DNA was purified from the mini-Mu unit integrants and hydrolyzed by the restriction enzymes *Rsa*I or *Sau*3A, many cleavage sites of which are located in the Mu unit, and one of them rather close to the Mu-**L** or Mu-**R** ends, respectively (Fig. S6). After circularization of the obtained DNA fragments via treatment with T4 DNA ligase at a low DNA concentration, inverse PCR was used with the divergent primer sets P31/P32 and P29/P30, which correspond to the internal portion of the Mu-**R** or Mu-**L** ends, respectively, as described earlier (Zimenkov et al. 2004). The sequence of the host DNA at its border with the mini-Mu unit was established via DNA sequencing of the obtained PCR fragments using the same primers.

### Shotgun cloning of the integrated mini-Mu unit

The chromosomal DNA of clone no. 10 was digested with the *Stu*I restriction endonuclease, which does not have any recognition sites in the integrated mini-Mu(**LER**)-YK unit. The obtained DNA fragments were cloned into the wide-host-range plasmid vector pCM110-Gm^R^ at its *Swa*I site (detailed construction scheme in Fig. S7), followed by transformation into *E. coli* TG1 cells and Km^R^ selection. Sequences adjacent to the Mu-**L** and Mu-**R** ends were determined by sequencing (Fig. S6).

## Results

### Designed elements of the Mu-driven transposition system for *C. glutamicum*

To investigate potential Mu-driven transposition in *C. glutamicum* cells, the previously developed dual-component system for gram-negative bacteria (Akhverdyan et al. 2011) was modified in the following fashion.

**Fig. 1:**
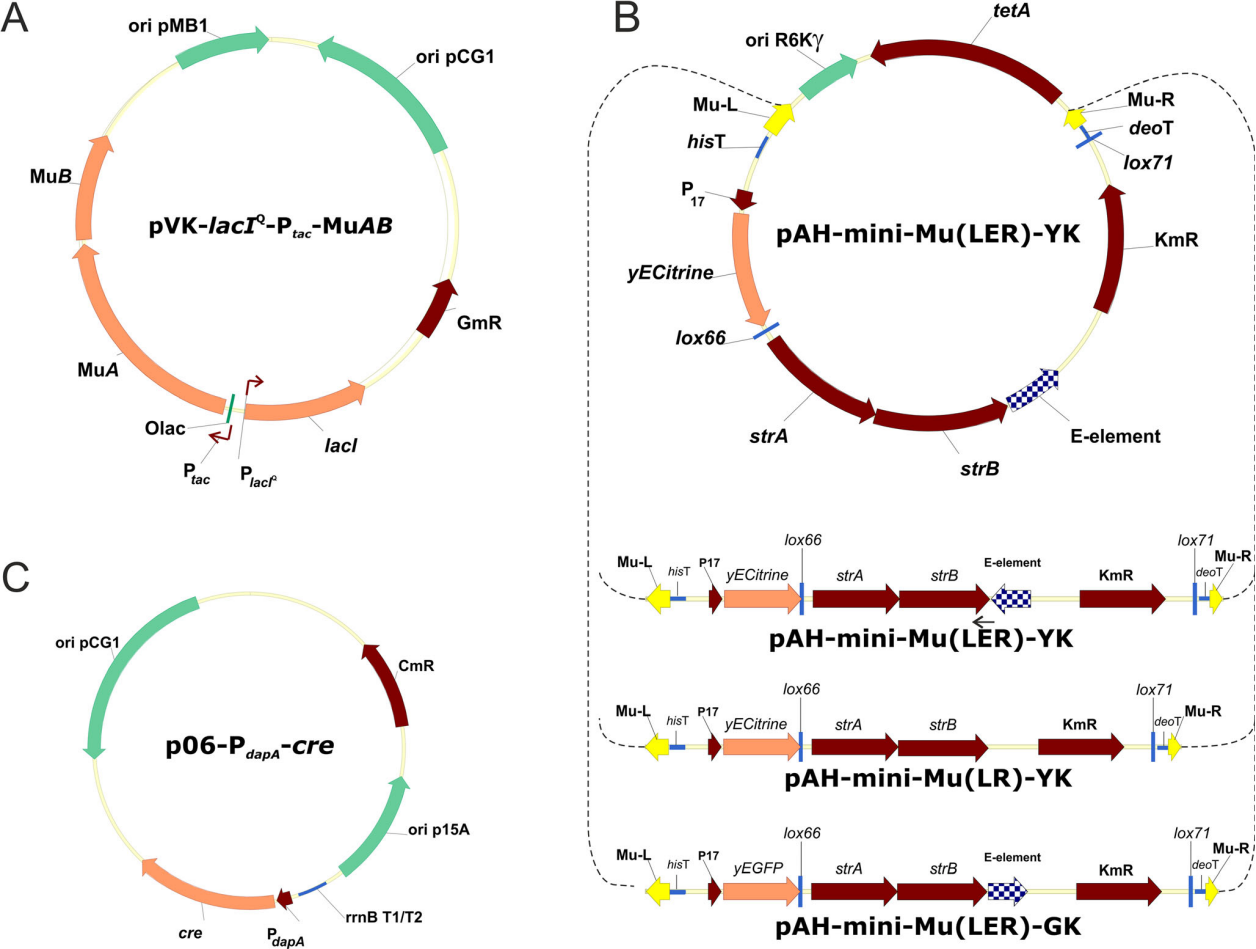
Schematic map of the mini-Mu-based plasmids: integration helper plasmid pVK-*lacI*^Q^-P_*tac*_-Mu*AB* (**a**); integrative plasmids pAH-mini-Mu(**LER**)-YK, pAH-mini-Mu($\mathbf{L}\overleftarrow{\mathbf{E}}\mathbf{R}$)-YK, pAH-mini-Mu(**LR**)-YK, and pAH-mini-Mu(**LER**)-GK (**b**); and excision helper plasmid p06-P_*dapA*_-*cre* (**c**)

First, the integration helper plasmid pVK9-*lacI*^Q^-P_*tac*_-Mu*AB* (Fig. 1a) was constructed for the expression of the MuA and MuB transposition factor genes in *C. glutamicum* cells. This plasmid was designed on the basis of the pVK9-Gm^R^ vector for rather stable maintenance in *C. glutamicum* cells, but with the ability to be cured under non-selective conditions (without the addition of Gm in the medium). Expression of the transposition factor genes Mu*AB* can be induced by IPTG addition via the introduction of P_*tac*_/O_*lac*_-promoter/operator region with the *lacI*^Q^ unit as their control element, which has been repeatedly used in *C. glutamicum* (Eggeling and Bott 2005; Kirchner and Tauch 2003; Nešvera and Pátek 2011; Ravasi et al. 2012). The Mu*AB* operon was cloned with the native ribosomal binding sites (RBSs) of its genes as no translation issues were anticipated in *C. glutamicum* according to calculations of RBS efficiency (https://salislab.net/software/) (Borujeni and Salis 2016). The *lacI*^Q^-P_*tac*_/O_*lac*_ system is known to have an inherently high basal level of transcription in non-induced conditions (Billman-Jacobe et al. 1994; Xu et al. 2010), and some problems with cloning toxic genes may occur. However, we did not experience any problems with the Mu*AB* genes.

As the second element of the Mu-driven transposition system, several integrative plasmids with mini-Mu units were constructed (Fig. 1b) using the conditionally replicated *pir*^+^-dependent (*oriR*γ) *E. coli* plasmid pAH162 (Haldimann and Wanner 2001; Posfai et al. 1994), which cannot autonomously replicate in *C. glutamicum* cells. The presence of Mu-**L**/**R** ends separates the integrative plasmids into two parts: non-Mu DNA and the mini-Mu unit. All integrative plasmids were named according to the specific features contained in their mini-Mu unit.

The non-Mu DNA of the plasmids carried the constitutively expressed gene *tetA* from Tn*10* (Hillen and Berens 1994; Lawley et al. 2000). To our knowledge, no experimental data concerning the expression of this *tetA* gene in *C. glutamicum* exists. Putative expression of this gene in *C. glutamicum* was very important for the confirmation of cointegrate formation due to nick-join-replicative transposition followed by its possible resolution, as the cointegrates and resolvants were detected by their Tc^R^ and Tc^S^ phenotypes, respectively. The results presented below confirmed rather low TetA activity in *C. glutamicum*; even though the expression level of *tetA* resulted in Tc^R^ to only 1 μg/mL of Tc in the medium, this resistance was higher than the basal resistance of the Tc^S^ control *C. glutamicum* strain.

The mini-Mu units carried the *strAB* genes, which were expressed at relatively low levels and conferred resistance to Sm at approximately 250 μg/mL. We expected that strains with several copies of the mini-Mu cassettes in the *C. glutamicum* chromosome could be selected using increased concentrations of Sm, as previously shown for *Methylophilus methylotrophus* (Abalakina et al. 2008). Additionally, all of the cassettes contained either the *yECitrine* or *yEGFP* gene-encoded mutant citrine or green fluorescent protein, respectively (Sheff and Thorn 2004). The mini-Mu cassettes mainly differed by the presence of an **E** element and its orientation towards the **L** and **R** ends. In the mini-Mu(**LR**) unit, the **E** element is absent; in the mini-Mu(**LER**) unit, the **E** element is properly arranged in relation to the **L**/**R** ends, as in the native Mu genome; and in the mini-Mu($\mathbf{L}\overleftarrow{\mathbf{E}}\mathbf{R}$) unit, the **E** element is located in the opposite direction (Fig. 1b).

*Lox66*/*lox71* sites (Albert et al. 1995) bracketed the DNA fragment containing the **E** element and Km^R^, Sm^R^ antibiotic-resistance markers, allowing irreversible excision of this fragment from the mini-Mu unit by the phage P1 Cre recombinase. For this purpose, an excision helper plasmid based on p06-Cm^R^ was constructed (Fig. 1c). Cre-mediated excision can occur due to the constitutive expression of the phage P1 gene encoding Cre, which was under the control of the *C. glutamicum* P_*dapA*_ promoter. This promoter of medium strength (Pátek 2005; Pátek et al. 1996) was used to avoid the overexpression of Cre, which could result in intrachromosomal Cre-dependent recombination not only between *lox*-like sites that are closely located in the same copy of the mini-Mu(**LER**) unit, but as well as between those in different chromosomally integrated units that are separated by long distances.

When successful excision of *lox*-bracketed DNA fragments occur, all mini-Mu units retain a mini-Mu(**LR**)-like form, consisting of only their antibiotic-markerless parts with expressed fluorescent protein gene, *yECitrine* or *yEGFP*, bracketed by Mu-**L**/**R** ends, which can be detected in the bacterial genome.

### Transposition of the mini-Mu(**LER**) unit from a superhelical integrative plasmid into the *C. glutamicum* chromosome

**Fig. 2:**
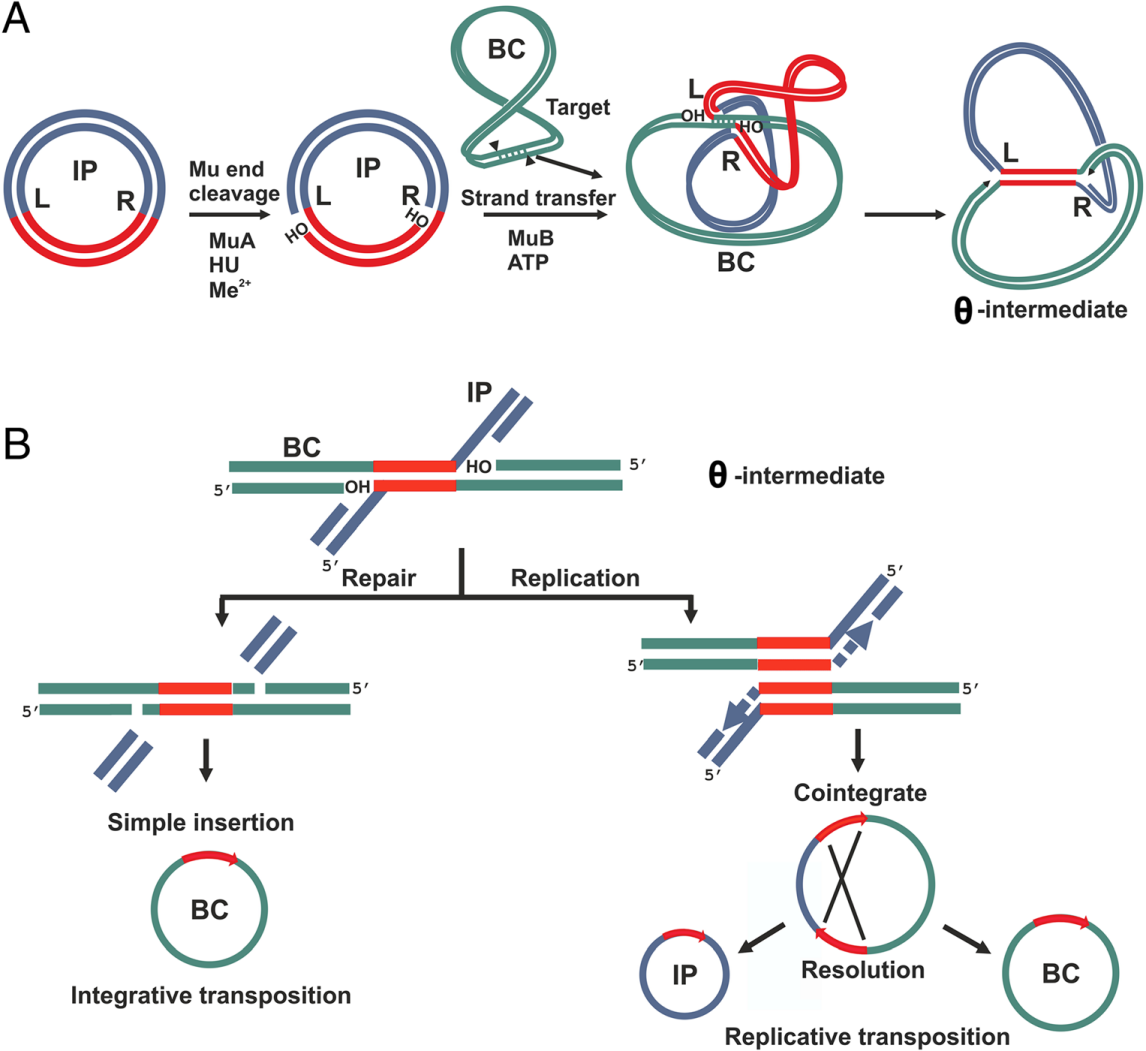
The two outcomes of Mu-driven DNA transposition from the mini-Mu unit-carrier integrative plasmid (IP) into bacterial chromosome (BC). On superhelical IP (supercoils not shown), in the presence of HU and divalent metal ions (Me^2+^), the transposase MuA generates endonucleolytic cleavages, producing 3′-OH nicks at Mu DNA **L**/**R** ends. Within the active site of MuA, in the subsequent strand-transfer step, the 3′-OH ends directly attack phosphodiester bonds in the target BC spaced 5 bp apart, Mu ends join to 5′-Ps in the BC, leaving 3′-OH nics on the target DNA, whose capture is promoted by MuB (**a**). The common θ intermediate can be resolved differently by the DNA repair/replication host-dependent machinery through reparative or replicative transposition pathways (**b**). The reparative transposition into the BC results in a “simple insertion” in which BC gains a copy of the mini-Mu unit. The replicative transposition, in turn, leads to a “cointegrate” formation in which I and BC fuse and two copies of the mini-Mu unit border this junction as direct repeats. The cointegrate can subsequently be resolved by homologous recombination between two mini-Mu units. Adapted from Akhverdyan et al. (2011) and Au et al. (2004)

To test potential Mu-driven transposition into the *C. glutamicum* chromosome, the integration helper plasmid-carrying strain *C. glutamicum* ATCC 13869[pVK-*lacI*^Q^-P_*tac*_-Mu*AB*] was initially obtained by electrotransformation (“Materials and methods”) of plasmid DNA and was then used as the recipient for electroporation with pAH-mini-Mu(**LER**)-YK, followed by the IPTG-induced expression of the Mu*AB* genes during cell cultivation (“Materials and methods”). Since the pAH-based integrative plasmid cannot replicate in *C. glutamicum* cells, the appearance of Km^R^ transformants putatively resulted from the integration of the mini-Mu unit into the host chromosome, through either the Mu-driven reparative or replicative transposition pathway (Fig. 2) (Craig 1996; Watson et al. 2004).

Based on previous experience with *M. methylotrophus* (Abalakina et al. 2008), the Sm^R^ levels may be lower for the Km^R^ clones obtained via the nick-join-reparative transposition pathway or for the rapidly resolved cointegrates that have only one copy of the integrated mini-Mu unit in the chromosome. For stable cointegrate formation, the entire integrative plasmid must be found in the bacterial chromosome of the Km^R^ clones, with two copies of the mini-Mu unit bracketed as a direct repeat of the bacterial and non-Mu plasmid parts of the cointegrate DNA; Sm^HR^ and Tc^R^ phenotypes could be detected for these clones.

**Fig. 3:**
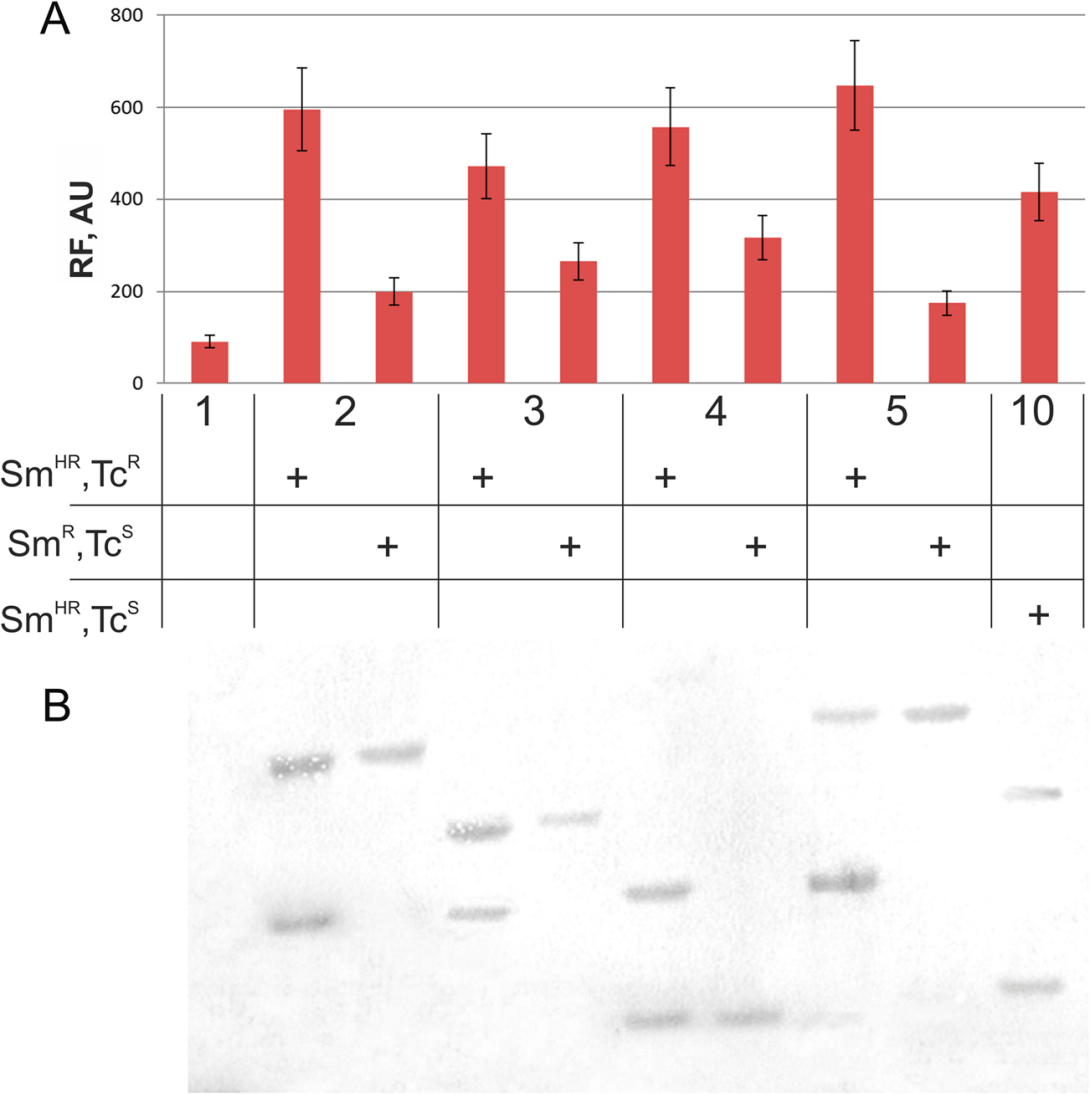
yECitrine relative fluorescence intensity (**A**) and Southern blot analysis (**B**) of the parental strain (**1**) independent co-integrants (Sm^HR^ and Tc^R^) and their resolvants (Sm^R^ and Tc^S^) (**2**, **3**, **4**, and **5**). For the Southern blot analysis, genomic DNA from the individual clones was digested with *Sma*I and hybridized with a *kan*-carrying DNA fragment amplified by PCR. (**10**) Results for clone no. 10, which had an unusual phenotype (Sm^HR^ and Tc^S^) after the standard Mu-driven integration procedure. Averages of two experiments are shown and in all cases standard deviation (SD) does not exceed 15%

The selection of transformants on media containing Km resulted in a set of Km^R^ clones that had a transformation frequency ≈ 1.6 × 10^−4^ (≈ 200 clones/100 ng DNA/1.2 × 10^6^ cells that survived after electroporation). This Mu-driven transposition efficiency was only tenfold lower than the transformation efficiency of the SH plasmid DNA (≈ 10^−3^) under these conditions. These transformants additionally manifested Sm^HR^ (95–99%) or Sm^R^ (1–5%) phenotypes on media supplemented with 750 or 250 μg/mL Sm, respectively. Moreover, practically all of the Sm^HR^ clones were resistant to the 1 μg/mL Tc that was added to the medium. At this stage, one clone (clone no. 10) was determined to have stable Sm^HR^ and Tc^S^ phenotypes (see below). As expected, all of the Sm^R^ clones were Tc^S^. The Sm^HR^ and Tc^R^ phenotypes of the obtained strains were rather stable: after five to eight generations, 97% of the single clones maintained this phenotype, and < 3% became Sm^R^ and Tc^S^, likely due to resolution of the cointegrate by an intrachromosomal general recombination process that resulted in the deletion and loss of the non-replicative pAH-based plasmid. Finally, the obtained Gm^R^ and Km^R^ strains of the integrants were cured of the helper plasmid by selecting for Gm^S^ and Km^R^ clones, as described earlier. Analysis of yECitrine-mediated fluorescence in the obtained Sm^HR^ clones and their Sm^R^ derivates confirmed our suppositions (Fig. 3A).

Southern hybridization experiments confirmed the nature of the obtained integrants. Chromosomal DNA from several Sm^HR^ and Tc^R^ clones and their Sm^R^ and Tc^S^ progenies was purified and hydrolyzed using the *Sma*I restriction endonuclease, which has a unique recognition site in the mini-Mu unit of the pAH-mini-Mu(**LER**)-YK plasmid that is located outside of the Km^R^ gene (see Fig. S8). After electrophoresis of the obtained DNA fragments in an agarose gel, Southern hybridization (“Material and methods”) was performed using the structural part of the Km^R^ gene, which was amplified by PCR in the presence of fluorescent oligonucleotide precursors, as a marker for the mini-Mu unit. All tested Sm^HR^ and Tc^R^ clones had two copies of the Km^R^ carrier mini-Mu unit in the bacterial chromosome, fully in accordance with their proposed cointegrate structure (Fig. 3B and its detailed explanation in Fig. S8). Moreover, all of the Sm^R^ and Tc^S^ derivatives retained only one copy of the mini-Mu unit at its initial point of integration: their hybridized DNA fragments consisted of the Km^R^ carrier part of the mini-Mu unit from the *Sma*I site in the mini-Mu to the nearest *Sma*I site in the bacterial chromosome, which is the same for the parental DNA and these derivatives. Furthermore, for the Sm^HR^ and Tc^R^ clones, the second hybridized DNA fragment was identical for all of the probes and corresponded to the *Sma*I-hydrolyzed, full-size pAH-mini-Mu(**LER**)-YK plasmid (Fig. S8).

On the basis of these experiments, the mini-Mu unit can be confidently concluded to transpose from the integrative SH plasmid into the *C. glutamicum* chromosome, mainly through the nick-join-replicative transposition pathway with the formation of a cointegrate. For a minor fraction (< 5%) of initially obtained integrants, we could not determine which of the two transposition pathways led to clone formation, reparative, which results in simple insertion, or replicative, which is accompanied by fast cointegrate resolution via general recombination.

According to the literature (Harshey 1983), MuA has no resolvase activity that could facilitate cointegrate resolution prior to finalizing the replication of the mini-Mu unit during the nick-join-replicative transposition process. So, cointegrate resolution is dependent only on host general recombinogenic activity, mainly on the activity of the *recA* gene product (Fitzpatrick et al. 1994). Thus, a RecA^−^ mutant of the *C. glutamicum* ATCC13869 strain was used as the recipient for the Mu-driven transposition of pAH-mini-Mu(**LER**)-YK, according to the standard protocol. The total efficiency of Km^R^ transformant formation in this experiment was (0.5 ± 0.2) × 10^−4^ (Km^R^ clones/100 ng plasmid DNA/surviving cells). The same with RecA^+^ isogenic recipient strain, approximately 97–98% of the obtained Km^R^ transformants manifested Sm^HR^ and Tс^R^ phenotypes, and the residual 2–3% were Sm^R^ and Tс^S^ for RecA^−^ strain. In contraposition to the Sm^HR^ and Tс^R^ cointegrates obtained in the Rec^+^ background, the phenotype of their Rec^−^ analogs was significantly more stable; at a minimum, Sm^R^ and Tc^S^ resolvants could not be detected under standard conditions (after 5–8 generations grown in non-selective conditions). Thus, the initial integrants that possessed Sm^R^ and Tc^S^ phenotypes in both the RecA^+^ and Rec^−^ strains were obtained mainly through nick-join-reparative transposition of the mini-Mu unit from the SH integrative plasmid into the *C. glutamicum* chromosome.

### Amplification of the mini-Mu(**LER**)-YK unit in the *C. glutamicum* chromosome

The data presented in Fig. 3 helped identify the nature of clone no. 10, which had stable Sm^HR^ and Tc^S^ phenotypes. The chromosome of clone no. 10 contained two copies of the Km^R^ gene that were likely obtained due to either (i) two independent mini-Mu(**LER**)-YK unit simple insertions resulting from reparative transposition from two integrative plasmids transformed into one recipient cell; (ii) two cointegrate resolutions obtained after consequent replicative transposition of mini-Mu units from two integrative plasmids into the chromosome; or, most likely, (iii) amplification due to intramolecular replicative transposition of the initially integrated mini-Mu(**LER**)-YK unit during growth of the bacterial cell with induced MuAB expression. In the latter case, the origin of the first integrated mini-Mu unit (through either reparative or replicative transposition coupled to cointegrate resolution) is not essential for the final conclusion of intrachromosomal mini-Mu unit amplification.

Each occurrence of intramolecular nick-join-replicative Mu-driven transposition is known (Craig 1996; Watson et al. 2004) to lead to (i) mini-Mu unit amplification, causing chromosomal inversion separated by inversely repeated mini-Mu units or (ii) deletion of non-replicative chromosomal DNA fragments due to the formation of two circular products, each carrying one copy of the mini-Mu unit: the first is capable of autonomous replication, while the second involves non-replicated parts of the bacterial DNA. Furthermore, if the circular DNA formed in the second instance consists of any essential gene(s) in a non-replicated part of bacterial DNA, only the fused circular DNA that results from the general recombination process between mini-Mu units in these two transposition products can be detected in the surviving clones possessing two directly repeated copies of mini-Mu units in their circular bacterial chromosomes (Fig. S9). Thus, the nature of this two-copied integrant (clone no. 10) could possibly be determined by investigating mini-Mu unit integration points. If two copies of the inversely repeated Mu units are located in random (independent) points of the chromosome, then two independent acts of mini-Mu unit integration must have occurred. However, inverse fragments of the *C. glutamicum* chromosome between two copies of inversely repeated mini-Mu units would unambiguously correspond to intrachromosomal Mu unit amplification. Directly repeated mini-Mu units in the chromosome could result either from two independent acts of unit integration or from intrachromosomal amplification of an initially integrated mini-Mu unit followed by fusion of two circular transposition products due to intermolecular general recombination between mini-Mu units.

Molecular cloning of chromosomal DNA fragment carrying the integrated mini-Mu unit was performed for clone no. 10 (“Materials and methods”). Among the plasmid DNA purified from three independently obtained Km^R^ *E. coli* transformants, we detected a plasmid with only one of two mini-Mu unit copies in a *Stu*I-hydrolyzed DNA fragment of approximately 11.2 kb that was bracketed by *C. glutamicum* DNA. Sequence analysis indicated that the host bordering DNA of the cloned mini-Mu unit corresponded to an inverted *C. glutamicum* genome structure. Additional confirmation of the estimated locations of both of the mini-Mu units was obtained via the application of a previously developed strategy (Zimenkov et al. 2004) based on inverse PCR with divergently oriented primers that correspond to the internal part of the Mu-**R** (or Mu-**L**) ends (data not shown). In addition, the locations of the host DNA that were linked by the two mini-Mu units were determined to be 484,726 and 2,370,010 bp according to the sequence of the *C. glutamicum* ATCC 13869 genome (GenBank AN AP017557.2). The detected structure of the cloned *Stu*I-fragment (Fig. S10) could only be obtained by the intramolecular nick-join-replicative Mu-driven amplification of an initially integrated mini-Mu unit via cointegrate formation, with inversion of the *C. glutamicum* ATCC13869 genome.

To provide artificial amplification, a *C. glutamicum* ATCC13869 strain carrying a single copy of the mini-Mu(**LER**)-YK unit in its chromosome was electrotransformed with the integration helper plasmid, followed by aerobic cultivation of a single transformant at 37 °C overnight in liquid BHI medium in the presence of Gm and IPTG with the final selection of Sm^HR^ variants. Sm^HR^ clones were detected at a frequency ≈ 5.0 × 10^−4^/cells, with several tens to hundreds of clones obtained in total (~ 10^5^ Sm^R^ and Gm^R^ cells). Notably, the detected efficiency of intrachromosomal amplification was three orders of magnitude lower than the efficiency of intracellular cointegrate formation of the already penetrated SH integrative mini-Mu(**LER**) unit-carrier plasmid with the bacterial chromosome of the *C. glutamicum* ATCC13869 strain (note the total frequency of cointegrant formation was ≈ 1.6 × 10^−4^, and the SH plasmid had to penetrate the cell (frequency with ≈ 1 × 10^−3^) before initiation of transpososome formation).

**Fig. 4:**
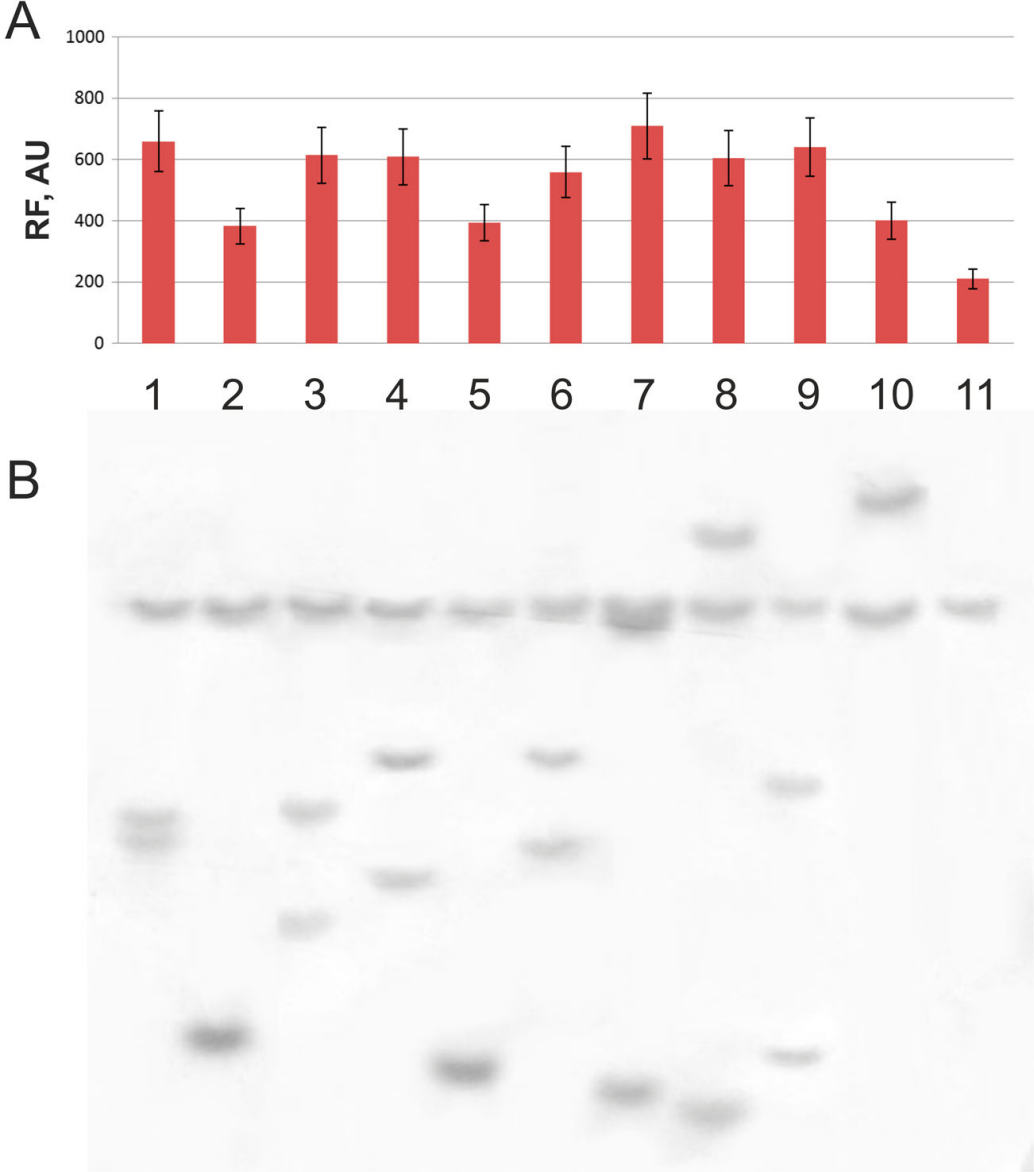
yECitrine relative fluorescence intensity (**A**) and Southern blot analysis (**B**) of clones selected as Sm^HR^ after mini-Mu(**LER**)-YK amplification (**1–10**) and parental clones with an initial single copy of the mini-Mu unit (**11**). For the Southern blot analysis, genomic DNA was isolated, digested with *Sma*I (which has a recognition site located in the non-*kan* part of the mini-Mu unit DNA) and hybridized with a *kan*-carrying DNA fragment amplified by PCR. Averages of two experiments are shown and in all cases standard deviation (SD) does not exceed 15%

In the final stage, the helper plasmid was cured from the obtained Sm^HR^ cells. Sets of Sm^HR^ clones were subjected to yECitrine-originated fluorescence analysis (“Materials and methods”). The fluorescence data and Southern hybridization results of the corresponding chromosomal DNA with the structural part of the Km^R^ gene from the mini-Mu(**LER**)-YK unit labeled are presented in Fig. 4A and B, respectively. The results confirmed that all of the tested clones were obtained via intramolecular Mu-driven replicative amplification of an initially integrated mini-Mu unit and ultimately contained up to two or three copies per chromosome.

### DNA superhelicity and the **E** element influence the efficiency of mini-Mu unit transposition from the integrative plasmid into the *C. glutamicum* chromosome

Three integrative plasmids, containing mini-Mu(**LER**)-YK, mini-Mu($\mathbf{L}\overleftarrow{\mathbf{E}}\mathbf{R}$)-YK, and mini-Mu(**LR**)-YK, were used to investigate the influences of plasmid superhelicity and the presence/location of the **E** element on the efficiency of Mu-driven transposition in the *C. glutamicum* chromosome. All plasmids were examined in both SH and covalently closed but relaxed forms (“Materials and methods”). The best integration efficiency was detected with SH plasmid DNA carrying the mini-Mu(**LER**) unit (Table 2). Relaxation of this plasmid decreased the efficiency of its transposition 1000-fold; nevertheless, this plasmid was still the best donor for transposition among the other relaxed plasmids. The plasmids with the mini-Mu(**LR**) unit demonstrated the lowest integration efficiency: for the SH plasmid, the transposition level was approximately 20-fold lower than that of the mini-Mu(**LER**) unit-carrying SH plasmid. Again, the relaxed form of the plasmid with the mini-Mu(**LR**) unit showed a 1000-fold decrease compared to its SH form. The plasmids with the mini-Mu($\mathbf{L}\overleftarrow{\mathbf{E}}\mathbf{R}$) unit manifested intermediate levels of integration efficiency. In its relaxed form, this plasmid had threefold lower integration efficiency than the relaxed plasmid with the mini-Mu(**LER**) unit, and it had approximately tenfold higher efficiency than the relaxed plasmid without the **E** element. At the same time, the plasmid with the mini-Mu($\mathbf{L}\overleftarrow{\mathbf{E}}\mathbf{R}$) unit in its SH form had an integration efficiency closer to its analog with mini-Mu(**LR**) than to others with mini-Mu(**LER**) units.

**Table 2:** An influence of the plasmid superhelicity (SH) and location of E element on the transposition efficiency

“Integrative” plasmid type	The efficiency of “integration” per 100 ng DNA	
	SH plasmid	Relaxed plasmid
pAH-mini-Mu(**LR**)-YK	(0.8 ± 0.2) × 10^−5^	(2.9 ± 0.6) × 10^−8^
pAH-mini-Mu(**LER**)-YK	(1.5 ± 0.5) × 10^−5^	(3 ± 1) × 10^−7^
pAH-mini-Mu(**LER**)-YK	(1.6 ± 0.4) × 10^−4^	(9 ± 3) × 10^−7^

### Mu-driven intrachromosomal amplification of different mini-Mu units in *C. glutamicum*

**Fig. 5:**
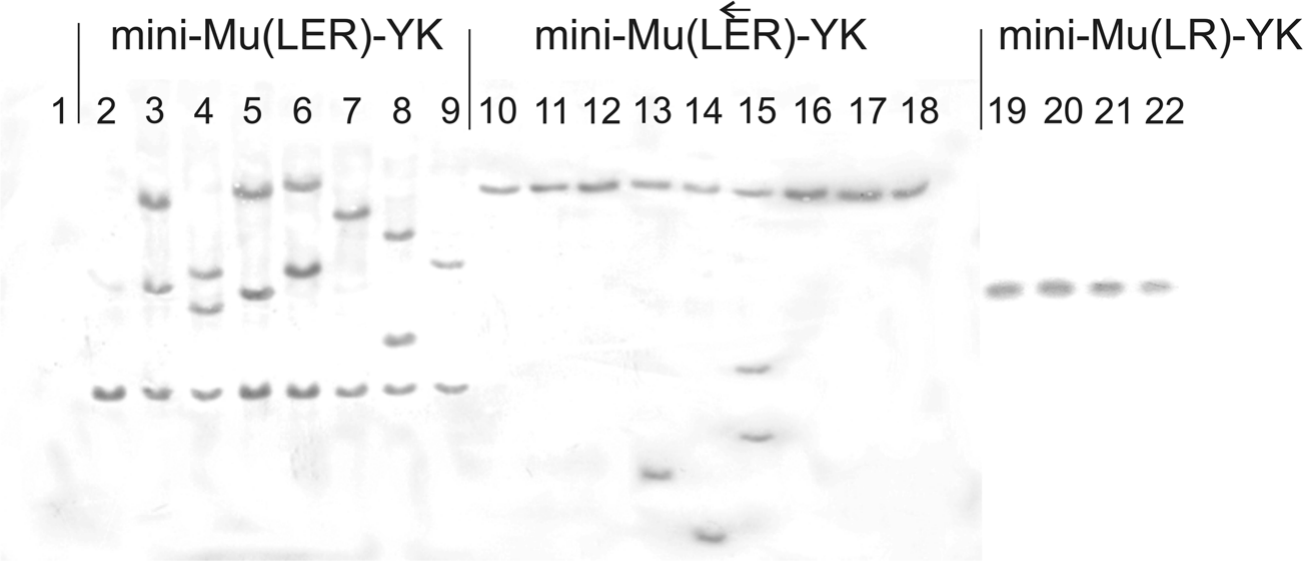
Southern blot analysis of the parental strain (**1**) and clones with a single integrated copy of a mini-Mu(**LER**)-YK unit (**2**), a mini-Mu($\mathbf{L}\overleftarrow{\mathbf{E}}\mathbf{R}$)-YK unit (**10**), and a mini-Mu(**LR**)-YK unit (**19**) as well as their independent derivatives obtained after growth with Mu*AB* expression (**3–9**), (**11–18**), and (**20–22**), respectively. For the Southern blot analysis, genomic DNA was digested with *Sma*I and hybridized with a *kan*-carrying DNA fragment amplified by PCR

Evaluating Mu-driven intrachromosomal amplification efficiency for mini-Mu units with converse orientations and/or the absence of an **E** element was interesting. For this, each of three different integrative plasmids in their SH forms was initially used to obtain corresponding resolved cоintegrates that had not lost the integration helper plasmid, pVK-*lacI*^Q^-P_*tac*_-Mu*AB*. For each strain, a single clone was grown in liquid medium with induced expression of Mu*AB,* and derivatives were selected at a high concentration of Sm. The efficiency of mini-Mu(**LER**) unit amplification was 4.0 × 10^−3^/cell (≈ 400 Sm^HR^ clones per 10^5^ Sm^R^ clones plated in total), which fully coincides with previous results. Approximately fivefold fewer Sm^HR^ clones were detected in a strain with mini-Mu($\mathbf{L}\overleftarrow{\mathbf{E}}\mathbf{R}$)-YK, and only a few Sm^HR^ clones were detected in a strain with mini-Mu(**LR**)-YK unit. Additionally, Southern hybridization confirmed the amplification of up to two to three copies of the Km^R^ gene in the chromosomes of Sm^HR^ clones with the mini-Mu(**LER**)-YK unit as well as several copies in Sm^HR^ clones with the mini-Mu($\mathbf{L}\overleftarrow{\mathbf{E}}\mathbf{R}$)-YK unit, but only one copy of the tested marker was maintained in the few Sm^HR^ clones with mini-Mu(**LR**)-YK (Fig. 5). Thus, an inverse orientation of the **E** element, $\overleftarrow{\mathbf{E}}$, can be concluded to result in a decreased amplification frequency of the corresponding mini-Mu units of approximately 6.0 × 10^−4^/cell. The absence of the **E** element in the mini-Mu(**LR**) unit structure decreased the frequency of the Mu-driven intramolecular replicative transposition to a level that was below 10^−5^, and amplification was not detected in the experiments with this type of unit.

### Consecutive independent integration/amplification/fixation of different mini-Mu(**LER**) units in the *C. glutamicum* chromosome

Earlier, an integration/amplification/fixation strategy for Mu-driven transposition of different mini-Mu(**LER**) units with excisable **E** elements was proposed on the basis of differences in the intrachromosomal transposition efficiencies of the mini-Mu(**LER**) and mini-Mu(**LR**) units detected in gram-negative bacteria (Akhverdyan et al. 2011). Data presented in the previous section serves as a background for testing the same strategy in *C. glutamicum*. One single-copy mini-Mu(**LER**)-YK-integrant of the *C. glutamicum* ATCC13869 strain (named 1YK) and two of its progeny obtained via Mu-driven amplification of this unit to two and three copies per chromosome (named 2YK and 3YK, respectively) were cured of the integration helper plasmid pVK-*lacI*^Q^-P_*tac*_-Mu*AB*. The internal parts of the intrachromosomal mini-Mu unit(s) bracketed by *lox66*/*lox71* sites and the included **E** element as well as the Km^R^ and Sm^R^ markers (Fig. 1b) were excised by the Cre recombinase (“Materials and methods”), followed by the elimination of the excision helper plasmid p06-P_*dapA*_-*cre* at the final stage of the experiment.

Targeted excision of the internal parts of the mini-Mu units was confirmed via the Km^S^ phenotype of the obtained strains. yECitrine-originated fluorescence was quantitatively evaluated for three parental mini-Mu(**LER**)-YK integrants, 1YK, 2YK, and 3YK, and for their Km^S^ derivatives with mini-Mu(**LR**)-Y type units (named 1Y, 2Y, and 3Y, respectively). The fluorescence data and subsequent Southern hybridization results probed with the structural part of the *yECitrine* gene confirmed the maintenance of the expected copy number of the truncated mini-Mu(**LR**)-like unit(s) after Cre-mediated chromosomal editing for all of the obtained strains (data not shown).

**Fig. 6:**
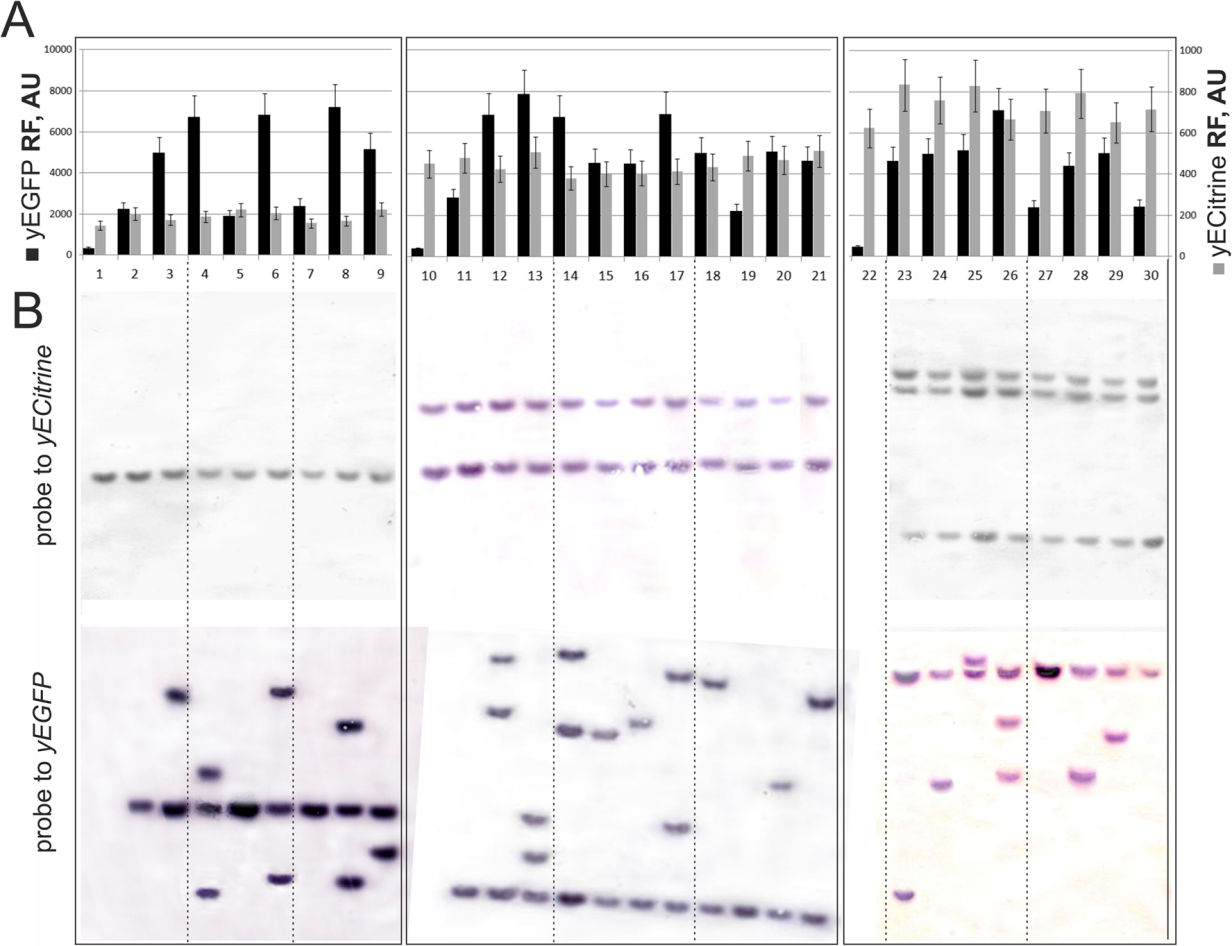
yECitrine and yEGFP relative fluorescence intensity (**A**) and Southern blot analysis (**B**) (*Sph*I restricted genomic DNA) using *yECitrine* or *yEGFP* as probes of a parental strain with a single mini-Mu(**LR**)-Y unit, 1Y **(1)**; a derivative of the 1Y clone with an introduced single mini-Mu(**LER**)-GK unit (**2**) and its derivatives with amplified mini-Mu(**LER**)-GK units (**3–9**); a parental strain with two mini-Mu(**LR**)-Y units, 2Y (**10**); a derivative of the 2Y clone with an introduced single mini-Mu(**LER**)-GK unit (**11**) and its derivative clones with amplified mini-Mu(**LER**)-GK units (**12–21**); a parental strain with three mini-Mu(**LR**)-Y units, 3Y (**22**, no Southern blot data); and a derivative of the 3Y clone with an introduced single mini-Mu(**LER**)-GK unit (**30**) and its derivative clones with amplified mini-Mu(**LER**)-GK units (**23–29**). *Sph*I has a unique recognition site in the mini-Mu unit structure that is beyond the *yECitrine* or *yEGFP* genes; the resulting step-by-step intramolecular amplification position of the hybridized bands in the Southern blots is retained in subsequent steps. Averages of three experiments are shown on graphs and in all cases SD do not exceed 15%

These three strains (*i*Y, where *i* = 1, 2, 3, indicating the number of the mini-Mu(**LR**)-Y units in the chromosome) were used for new Mu-dependent integration followed by amplification using the mini-Mu-(**LER**)-GK unit, which differs from the previously used mini-Mu-(**LER**)-YK in that it contains the *yEGFP* gene rather than the *yECitrine* gene. All of the procedures used for Mu-driven nick-join-replicative transposition were performed according to the designed and described protocols above. At both transposition stages, after integration and amplification, the obtained strains were evaluated using both yECitrine and yEGFP-originated fluorescence as well as Southern hybridization with the structural parts of the *yECitrine* and *yEGFP* genes as markers (Fig. 6).

All of the data confirmed that integration followed by amplification leading to one to three copies of the mini-Mu(**LER**)-GK unit in the chromosomes of all three recipients used was successfully realized, and a set of double *C. glutamicum* integrants *i*Y-*j*GK (*i*, *j* = 1, 2, 3, indicating the number of *yECitrine*- and *yEGFP*-gene carrier units in the chromosome, respectively) was obtained. Moreover, the chromosomal positions of the mini-Mu(**LR**)-Y units during Mu-driven intrachromosomal amplification of the mini-Mu(**LER**)-GK units were maintained (fixed).

## Discussion

Mu-driven replicative transposition is a highly efficient, convenient method for recombinant DNA integration and amplification in plasmid-less industrial strains that are based on gram-negative bacteria (Akhverdyan et al. 2011). This method is especially relevant and useful for organisms without developed powerful and comprehensive genetic tools for chromosomal editing. Considering the possible inclusion of a Mu-based integration/amplification strategy in a set of genetic tools for gram-positive microorganisms of industrial interest, in particular, we decided to modify a previously developed dual-component plasmid system (Akhverdyan et al. 2011) for expression in different strains of *Corynebacterium glutamicum*.

In this study, the *C. glutamicum* ATCC13869 strain was tested for Mu-driven transposition. Replicative transposition of the mini-Mu(**LER**) unit through cointegrate formation was confirmed as the main pathway of mini-Mu unit integration (> 95%) into the bacterial chromosome from the SH integrative plasmid. It was interesting to note that efficiency of initial transposition and intrachromosomal mini-Mu(LER) unit amplification were significantly increased if *C. glutamicum* cells were grown at 37 °C before inducing MuAB transposition factors as described in “Materials and methods.” Probably, this effect could be based on the possible synthesis of some “heat-shock proteins” and chaperones facilitated the transposition.

As shown from the results obtained for the isogenic *C. glutamicum* ATCC13869-(*recA*^−^) strain, a minor fraction of the *recA*^+^ strain integrants (< 5%) was likely obtained via nick-join-reparative transposition. Whether host proteins participate in this pathway of transposition in *C. glutamicum* is unknown; however, direct analogs of the *E. coli* RecBCD nuclease that collaborates with the transpososome in the repair of simple Mu insertions (Choi et al. 2014) are absent in this bacterium (Nakamura et al. 2003).

Both *E. coli* Mu-mediated reparative and replicative transposition pathways are known to be catalyzed by a higher order DNA protein complex called the transpososome, which is organized by bridging interactions among three DNA sites, the **L**/**R** ends of Mu and the **E** element. This complex is mediated by six subunits of the MuA transposase and assisted by the host proteins HU and IHF to form an **LER** synapse (Harshey 2014). The presence of the **E** element in the native transpososome **LER** structure increases the efficiency of transposition over two orders of magnitude in vivo (Leung et al. 1989) and accelerates the initial rate of transposition in vitro by a similar amount (Surette et al. 1989). In *E. coli*, the site-specific IHF-mediated bending of DNA at the **E** element located in the *cis* orientation with respect to the **L**/**R** ends facilitates efficient transposition of the mini-Mu(**LER**) unit, especially when the SH density (σ) of the transposed DNA becomes low (Surette and Chaconas 1989). To our knowledge, the presence of functionally active DNA-binding analogs of the *E. coli* IHF and HU proteins have not been reliably identified among corynebacterial proteins; however, a gene encoding the putative integration host protein cIHF (GenBank accession number: CG1811 (CorglutaCyc)) was annotated in the *C. glutamicum* ATCC 13032 genome (Kalinowski et al. 2003).

In the present study, the dependence of Mu-driven transposition efficiency in *C. glutamicum* on the presence of the **E** element in mini-Mu units and on the superhelicity of integrative plasmids was tested. A 20-fold difference in transposition efficiency was observed for the SH integrative plasmids, and the mini-Mu(**LER**) unit with properly located **E** element had the highest yield of transformants (Table 3) compared to the mini-Mu(**LR**) unit-carrying SH plasmid without **E**, which demonstrated the lowest yield. The plasmid with the mini-Mu($\mathbf{L}\overleftarrow{\mathbf{E}}\mathbf{R}$) unit had a transposition efficiency tenfold lower than that of the mini-Mu(**LER**) unit and only twofold higher than that of the mini-Mu(**LR**) unit. For rather small integrative plasmids, due to the close spatial location of the Mu-**L**/**R** ends in the SH DNA structure, the process of minimal transpososome formation occurred rather efficiently even without **E** element facilitation. However, inversely located enhancer, $\mathbf{L}\overleftarrow{\mathbf{E}}\mathbf{R}$, in the corresponding SH plasmid had insufficient structural freedom to significantly increase the efficiency of full-sized transpososome assembly.

**Table 3:** The number of Sm^HR^ clones, selected on the high concentration of Sm (0.75; 1.0 μg/mL) after mini-Mu unit amplification

Sm μg/mL	The amount of seeded Sm^R^ cells	mini-Mu(**LR**)-YK		mini-Mu(**LER**)-YK		mini-Mu(**LER**)-YK	
		Control	Amplification	Control	Amplification	Control	Amplification
0.75	10^5^	–	13 ± 7	7 ± 5	74 ± 19	8 ± 5	415 ± 57
1.0	10^5^	–	–	2 ± 1	46 ± 2	3 ± 2	241 ± 17

Based on the data obtained in the present study, Mu-driven transposition from the relaxed form of the integrative plasmid could be a closed experimental model of intrachromosomal replicative amplification under conditions where DNA-binding proteins constrain supercoils in bacterial DNA and significantly decrease chromosomal SH density (Dillon and Dorman 2010). Indeed, the estimated electroporation/penetration efficiency of the *C. glutamicum* ATCC 13869 strain with SH plasmid DNA was ≈ 1 × 10^−3^/100 ng DNA/surviving cells. At the same time, the efficiency of transposition (penetration + cointegrate formation) of the mini-Mu(**LER**) unit-carrying relaxed plasmid into the same strain was ≈ 9 × 10^−7^/100 ng DNA/surviving cells. Thus, the efficiency of intracellular cointegrate formation between the relaxed integrative plasmid and the *C. glutamicum* chromosome was estimated to be approx. 9 × 10^−4^/cell. This estimation is very close to the experimentally detected efficiency of mini-Mu(**LER**) unit intrachromosomal amplification, which was ≈ 5 × 10^−4^/cell. This assumption was additionally confirmed by the detection of threefold decreases in the transposition efficiency of relaxed integrative plasmids with the mini-Mu($\mathbf{L}\overleftarrow{\mathbf{E}}\mathbf{R}$) unit compared to the analogous mini-Mu(**LER**) unit-carrying plasmid, which correlated well with the twofold difference in the intramolecular amplification level detected for the corresponding mini-Mu units.

The results obtained for the mini-Mu(**LR**) unit were rather interesting and not easily predicted. Cointegrate formation between the relaxed integrative plasmid with the mini-Mu(**LR**) unit and the *C. glutamicum* chromosome occurred with a tenfold lower transposition efficiency than the mini-Mu($\mathbf{L}\overleftarrow{\mathbf{E}}\mathbf{R}$) unit-carrying relaxed plasmid. At the same time, the difference in the frequencies of intramolecular amplification of the mini-Mu(**LR**) and mini-Mu($\mathbf{L}\overleftarrow{\mathbf{E}}\mathbf{R}$) units was significantly higher, and this amplification could not be detected for the mini-Mu(**LR**) unit under the experimental conditions. This formation of the transpososome structure resulted in Mu end pairing without participation of the **E** element, which presented no significant difficulties for the relaxed integrative plasmid substrate, but was a serious problem when the constrained bacterial chromosome served as the substrate. Notably, the centrally located strong gyrase binding site (**SGS**) is required for efficient synapsis and formation of the transpososome, which is the obligatory first step for the initiation of Mu DNA replication in the whole Mu prophage genome, even though it carries the native **E** enhancer element. DNA gyrase bound at the **SGS** site allows the rapid, efficient synapsis of Mu prophage **L**/**R** ends within the constraints imposed by the structure of the bacterial nucleoid, doing so by promoting the formation of a supercoiled loop, with the apex site and prophage ends synapsed at the base of the loop (Pato 2004; Pato and Banerjee 2000).

The dramatic decrease in the intramolecular transposition efficiency of mini-Mu(**LR**) units compared to the mini-Mu(**LER**) units located in the restrained protein-bound DNA of the *C. glutamicum* chromosome allowed the application of a genome modification strategy previously developed for the *Methylophilus methylotrophus* AS-1 (Akhverdyan et al. 2011) strain. A consecutive independent integration/amplification/fixation process via excision of the **E** element in different mini-Mu(**LER**) units in the *C. glutamicum* chromosome was successfully demonstrated in the present study.

Note that application of the pVK9-*lacI*^Q^-P_*tac*_-Mu*AB* helper plasmid resulted in amplification of the initially transposed mini-Mu(LER) unit up to two to three copies. At the same time, using the analogous helper plasmid with decreased expression level of Mu*AB* due to their transcription with “weaker” promoter led to only one additional copy of mini-Mu (data not shown). So, amplification events could be controlled by the expression level of Mu*AB*.

Additionally, Mu-driven replicative integration with the formation of cointegrate structure, followed by its resolution, was demonstrated using the same two-plasmid system in the widely used, wild-type lab strain of *C. glutamicum*, ATCC13032, and its prophage-free derivative MB001, which has also applications in biotechnology (Baumgart et al. 2013). The efficiency of initial Mu-driven integration (Km^R^ clones/100 ng SH plasmid DNA/surviving cells) provided by optimized electrotransformation conditions for each strain was ≈ 10^−7^ for ATCC13032 (≈ 200 Km^R^ integrants/100 ng plasmid DNA/2.5 × 10^9^ surviving cells) and 10^−6^ for MB001.

**Fig. 7:**
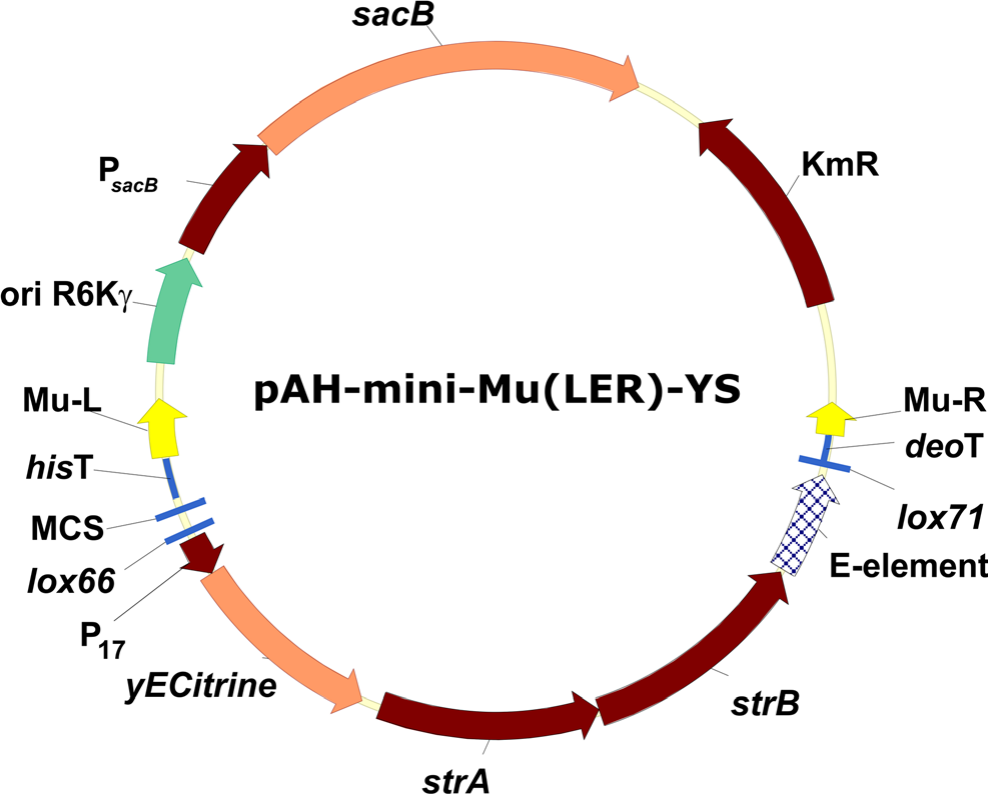
Scheme of the new integrative plasmid vector pAH-mini-Mu(**LER**)-YS (GenBank accession no. MG014200)

To widely use this confirmed Mu-mediated transposition system as a useful tool for chromosomal editing in *C. glutamicum*, the more convenient mini-Mu(**LER**)-type plasmid, pAH-mini-Mu(**LER**)-YS (Fig. 7), was designed as a potential vector for cloning genes of interest. Application of the dependable Km^R^ marker, located in the non-Mu DNA part of this plasmid, is more convenient for the selection of cointegrates than the Tc^R^ marker. However, the expressed *Bacillus subtilis sacB* gene can be used as a counter-selective marker and facilitates the detection of *C. glutamicum* resolvants in sucrose-containing media (Jäger et al. 1992). Application selection on 20% sucrose-containing medium resulted in 15–30% of colonies with resolved structure among all colonies against 2–3% in case of pAH-miniMu(LER)-YK. The Sm^R^ marker located in the mini-Mu unit of this new plasmid can be used for the direct selection of integrants, followed by the possible selective intrachromosomal amplification of the integrated (**LER**)-like unit. Moreover, the presence of the *yECitrine* gene can help to semi-quantitatively estimate the obtained mini-Mu unit copy number in the selected Sm^HR^ clones by fluorescence. All of the technical facilitating genes (E element, Sm^R^, and *yECitrine*) can be excised from the integrated mini-Mu units in a Cre-dependent fashion due to the proper location of *lox66/71* sites, and only the small markerless part of the vector plasmid containing the gene(s) of interest cloned into its multiple cloning site (MCS) is retained. This plasmid could be used as an integrative vector for cloning, followed by Mu-driven transposition in the chromosomes of different organisms and in *C. glutamicum*, in particular. Up to date, the maximal 8-kb target DNA fragments were successfully inserted and amplified in our lab via pAH-mini-Mu(**LER**)-YS–like vector.

## Electronic supplementary material

ESM 1 (PDF 1254 kb)
