# Supplementary material for: Mu-driven transposition of recombinant mini-Mu unit DNA in the Corynebacterium glutamicum chromosome
Source: Appl Microbiol Biotechnol. 2018 Feb 1;102(6):2867–84. doi: 10.1007/s00253-018-8767-1 (PMC5847225; doi:10.1007/s00253-018-8767-1)
Supplement: Supplementary file 1 — (PDF 1254 kb) [file 253_2018_8767_MOESM1_ESM.pdf]

APPLIED MICROBIOLOGY AND BIOTECHNOLOGY

Mu-Driven Transposition of Recombinant mini-Mu unit DNA in *Corynebacterium glutamicum* chromosome

Natalya V. Gorshkova, Juliya S. Lobanova, Irina L. Tokmakova, Sergey V. Smirnov, Valerii Z. Akhverdyan, Alexander A. Krylov, and Sergey V. Mashko♣

Ajinomoto-Genetika Research Institute,

1-st Dorozhny proezd, 1-1, 117545 Moscow, Russian Federation

♣Corresponding author - E-mail addresses: sergey_mashko@agri.ru

Tel: +7 (495) 780-3378 ext. 511

Fax: +7 (495) 315-0640

Supplementary materials

Table S1 Oligonucleotides used in this study

Primer	Sequence (5' – 3')[a]	Application / Specific
P1	GGATTCATGCAAGGAAACTATCATGCAGGTGACACCCTATCATTG	Construction of pAH-mini-Mu(**LR**)-YK
P2	CGTACTTCAAGTGAATCAATACAACCCCCATTCACTGCCAGAGC	Construction of pAH-mini-Mu(**LR**)-YK
P3	GAAAAGATTACTTCGCGTGAACGTTTTTTGAAGCTGTTAT	Construction of pAH-mini-Mu(**LR**)-YK
P4	CTAGTCGCTGAATATTCCTTTTG	Construction of pAH-mini-Mu(**LR**)-YK
P5	GCCCTTCCCAACAGTTGCAATTCTTAACGACGGTAATTG	Construction of pAH-mini-Mu(**LR**)-YK
P6	CAGCTTCAAAAAACGTTCACGCGAAGTAATCTTTTC	Construction of pAH-mini-Mu(**LR**)-YK
P7	ATATCTAGAATCTATCGATAATGATGACATCGCTGGTTAG	Construction of pAH-mini-Mu(**LR**)-YK / *Xho*I, *Cla*I underline
P8	CAATTACCGTCGTTAAGAATTGCAACTGTTGGGAAGGGC	Construction of pAH-mini-Mu(**LR**)-YK
P9	CTGACATGGGAATTAGCCATGTAACTAGCTAGTCGCTGAATATTCCTTTT	Construction of pAH-mini-Mu(**LR**)-YK
P10	ATATCTAGAATCTATCGATAATGATGACATCG	Construction of pAH-mini-Mu(**LR**)-YK / *Xho*I-*Cla*I underline
P11	CATTTTTCTCTCCAGCTTTCAAGATCCCCATGTAATGAATAAAAAG	Construction of pAH-mini-Mu(**LR**)-YK
P12	TAGTTTCCTTGCATGAATCCATA	Construction of pAH-mini-Mu(**LR**)-YK
P13	TTACATGGGGATCTTGAAAGCTGGAGAGAAAAATG	Construction of pAH-mini-Mu(**LR**)-YK
P14	GCGATAAGAGTAATTGTGTTTCGC	Construction of pAH-mini-Mu(**LR**)-YK
P15	*ATAACTTCGTATAATGTATGCTATACGAACGGTA*GCGATAAGAGTAATTGTGTTT CGC	Construction of pAH-mini-Mu(**LR**)-YK / lox71 mark *italic*
P16	CAATGATAGGGTGTCACCTGCATGATAGTTTCCTTGCATGAATCC	Construction of pAH-mini-Mu(**LR**)-YK
P17	ATAACTTCGTATAATGTATGCTATACGAAC	Construction of pAH-mini-Mu(**LR**)-YK
P18	ATAGGGCCCGATTCCAGTGCGATTTTGATCATCTGCATCCTTAGTTCCTATTCCG AAGTTC	Construction of pAH-mini-Mu(**LR**)-YK / *Apa*I underline
P19	ATATCTAGATATCCCGGGTTGCAGCATTACACGTCTTGAGC	Construction of pAH-mini-Mu(**LR**)-YK / *Xho*I, *Sma*I underline

P20	ATACTCGAGGAACTGCACATTCGGGATATTTCTC	Construction of pAH-mini-Mu(**LR**)-YK / *Xho*I underline
P21	ATACCCGGGCCATCTAGTATGACGTCTGTCGCACC	Construction of pAH-mini-Mu(**LR**)-YK / *Sma*I underline
P22	ATACTCGAG*ATAACTTCGTATAGCATACATTATACGAACGGTA*TTATTTGTACAATTCATCAATACCATGG	Construction of pAH-mini-Mu(**LR**)-YK / *Xho*I underline, lox66 mark *italic*
P23	ATAATCGAT*AGGAGGT*TAATTAACATGTCTAAAGGTGAAG	Construction of pAH-mini-Mu(**LR**)-YK / *Cla*I underline, SD mark *italic*
P24	ATACCCGGGGCATCGACATCACATCGTATTCAAC	Construction of pAH-mini-Mu(**LER**)-YK, pAH-mini-Mu(**LĒR**)-YK / *Sma*I underline
P25	ATACCCGGGACATTTAAAAACCCTCCTAAGTTTTG	Construction of pAH-mini-Mu(**LER**)-YK, pAH-mini-Mu(**LĒR**)-YK / *Sma*I underline
P26	TTGCAGCATTACACGTCTTGAGC	Construction of pAH-mini-Mu(**LER**)-YK, pAH-mini-Mu(**LĒR**)-YK
P27	GGGCCATCTAGTATGACGTCTGTC	Construction of pAH-mini-Mu(**LER**)-YK, pAH-mini-Mu(**LĒR**)-YK
P29	TTTCGTACTTCAAGTGAATCAATACA	Detection of integration site MuattL- 5'(-)
P30	GGAGGACATTGGATTATTCGG	Detection of integration site MuattL-5'(+)
P31	TTTAGCTTTCGCGCTTCAAATG	Detection of integration site MuattR-5'(+)
P32	TTTATCGTGAAACGCTTTCGC	Detection of integration site MuattR-5'(-)
P33	ATACTGCAGATGATCAAAATCGCACTG	Detection of integration site (attR)
P34	ATAGAATTCTTAACGACGGTAATTGAG	Detection of integration site (attL)
P35	ATAGCTAGCAGCGGGTGATGGGACTAACG	Construction of pCM110-Gm^{R}
P36	ATAGCTAGCTCTCTGTGCATGGTGAAAACGG	Construction of pCM110-Gm^{R}
P39	ATATATGCTAGCAGCGGGTCATGGGACTCACGCCTGAGCCTAGCAGCGGGTGATGG	Construction of pVK-*lacI*^{Q}-P$_{tac}$-Mu*AB* / *Nhe*I underline
P40	ATATATGCTAGCTCTCTGTGCATGGTGAAAACGG	Construction of pVK-*lacI*^{Q}-P$_{tac}$-Mu*AB* / *Nhe*I underline
P41	ATATCTAGA*GTGAAATTGTTATCCGCTCACAATTCCACACATACGAGCCGATGATTAATTGTCAACAGCTC*ATTAATTCAGAATATACACCATCGAATGG**T**GCAAAACC	Construction of pVK-*lacI*^{Q}-P$_{tac}$-Mu*AB* / P$_{tac}$ mark *italic*, Q mutation- mark bold, *Xba*I underline
P42	ATAGGTACCAGCTAACTCACATTAATTGCGTTGC	Construction of pVK-*lacI*^{Q}-P$_{tac}$-Mu*AB* / *Kpn*I underline
P43	ATAGTCGACAGGAGGTGTTAAATGTCCAATTTACTGA	Construction of p06-P$_{dapA}$-*cre* / *Sal*I underline
P44	ATAGGTACCCATAAATATCAAATAATTATAGC	Construction p06-P$_{dapA}$-*cre* / *Kpn*I underline
P63	ATAATCGAT*AGGAGG*AACCACCATGTCTAAAGGTGAAGAATTATTC	Construction of pAH-mini-Mu(**LER**)-GK / ClaI underline, SD mark *italic*
P64	ATACTCGAG*ATAACTTCGTATAGCATACATTATACGAACGGTA*GCTTATTTGTACAATTCATCCATACC	pAH-mini-Mu(**LER**)-GK / *Xho*I underline, lox66 mark *italic*
P37	TGATTGAACAAGATGGAT	Km^{R} gene (test and for hybridization probe)
P38	CTCAGAAGAACTCGTCAA	Km^{R} gene (test and for hybridization probe)
P56	ATAATAGCTAGCTGATGCTCGATGAGTTTTTTC	Construction of pVK-*lacI*^{Q}-P$_{tac}$-Mu*AB* / *Nhe*I underline
P57	ATATATGCTAGCTGTAAAAAACTTCTGCGTCGCCCCGCAAATTTTCG	Construction of pVK-*lacI*^{Q}-P$_{tac}$-Mu*AB* / *Nhe*I underline

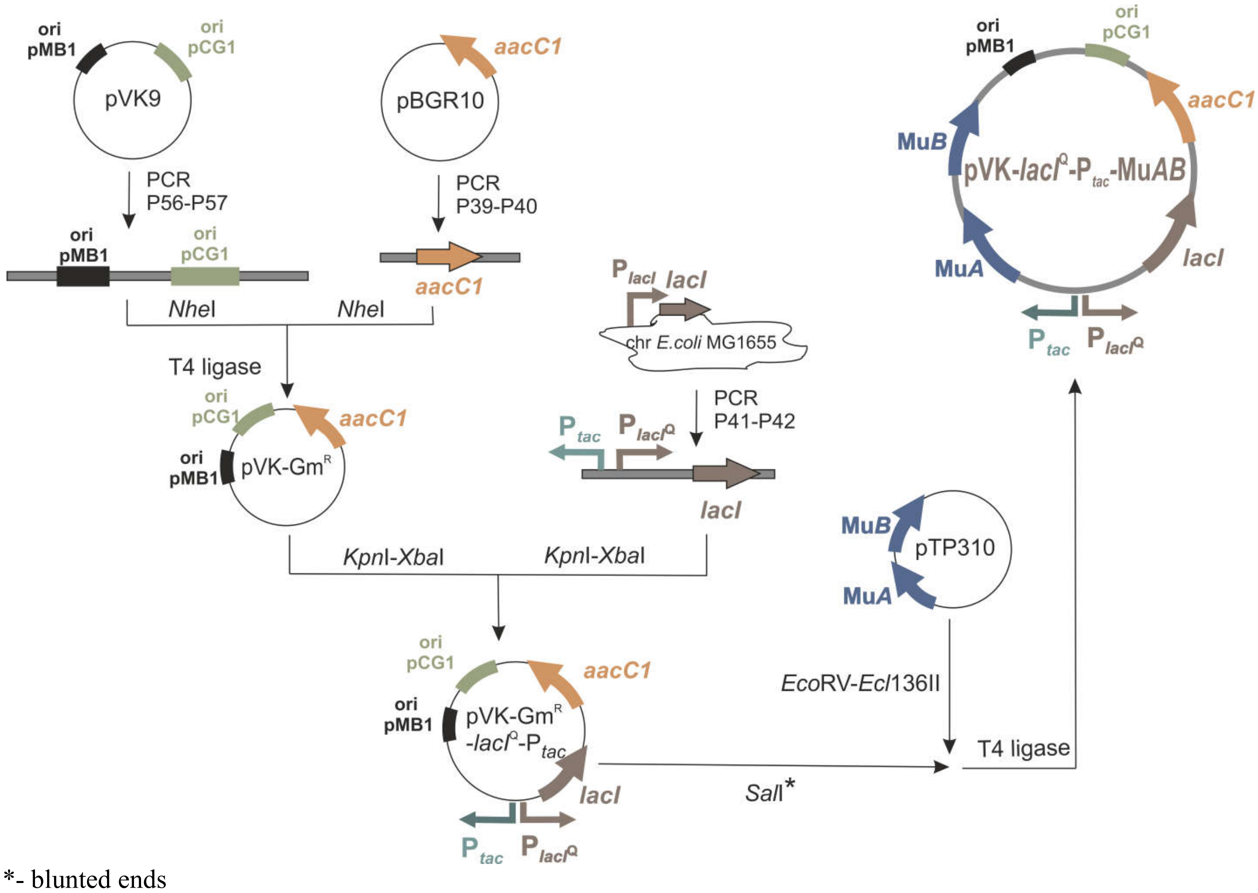

*- blunted ends

Figure S1 Scheme of construction integration helper plasmid pVK-*lacI*^{Q}-P$_{tac}$-Mu*AB* (GenBank ANo MG014199)

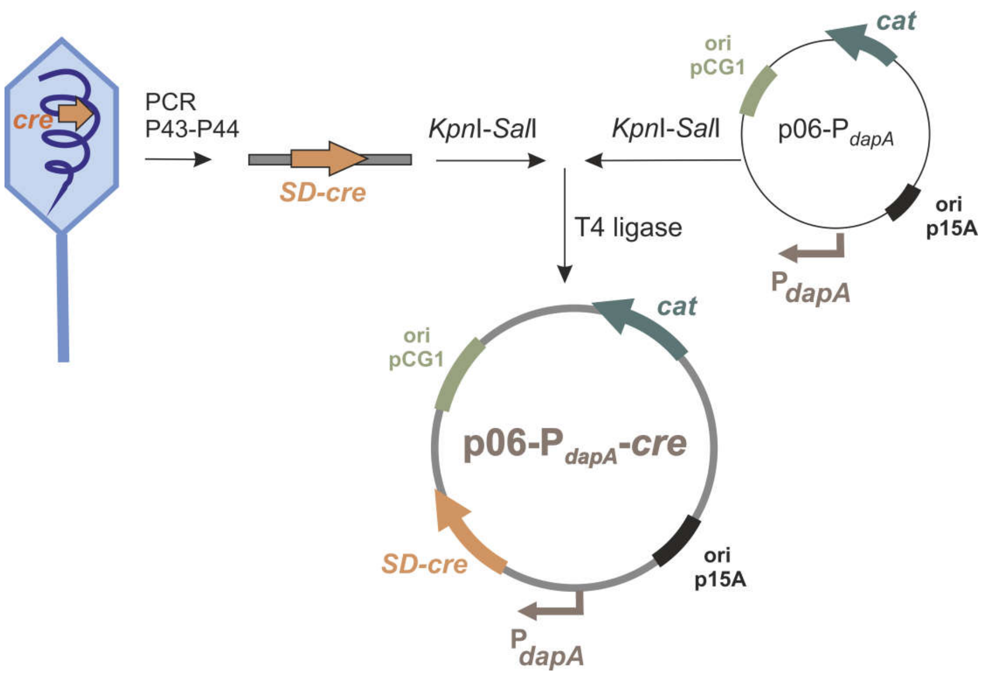

Figure S2 Scheme of construction excision helper plasmid p06-P_{dapA}-*cre* (GenBank ANo MG014197)

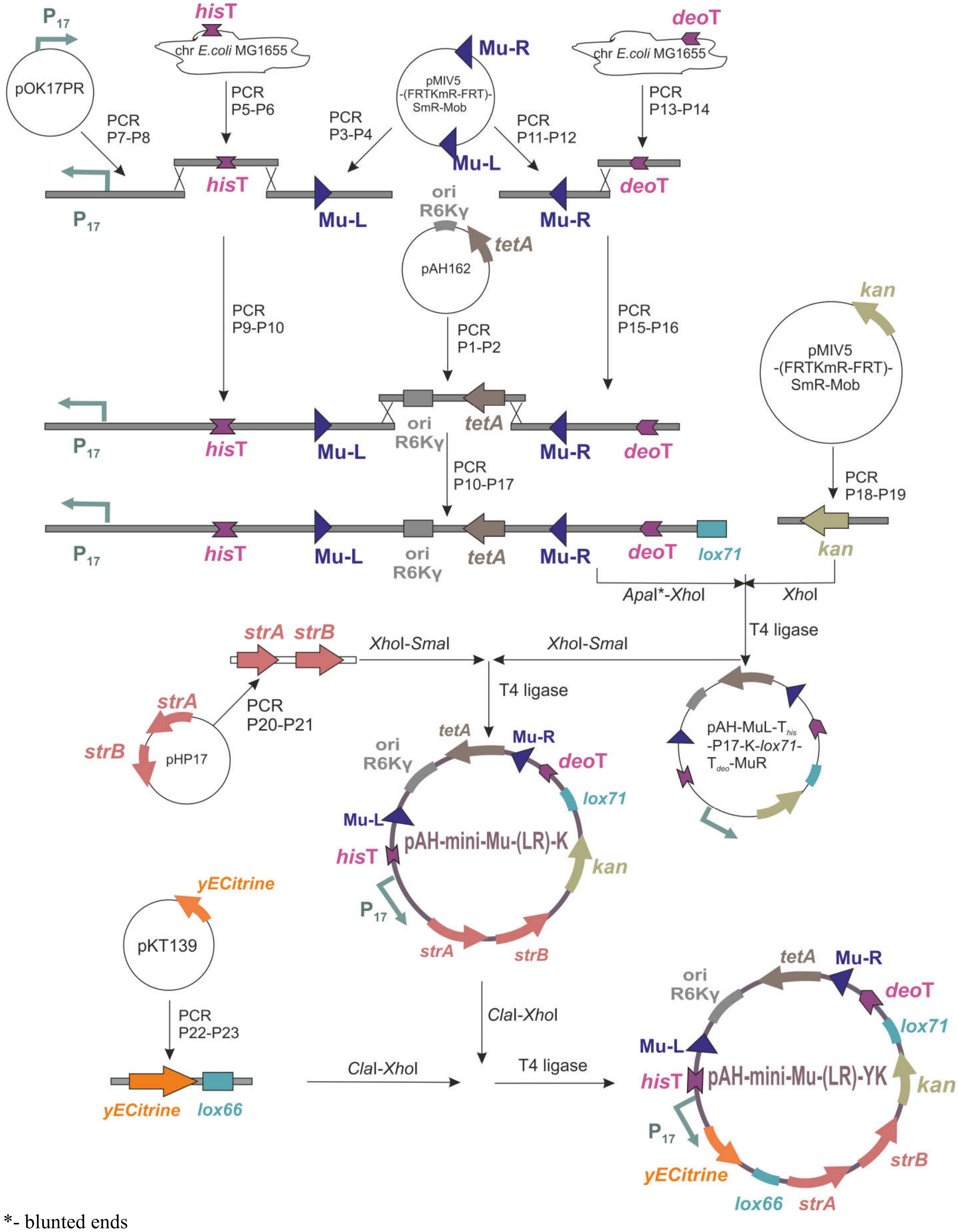

*- blunted ends

Figure S3 Scheme of construction “integrative” plasmid pAH-mini-Mu(**LR**)-YK. OE-PCR and restriction-ligation cloning were used for construction of “integrative” recombinant plasmid with mini-Mu units. This plasmid was used as the basic for construction of the set of analogous “integrative” plasmids

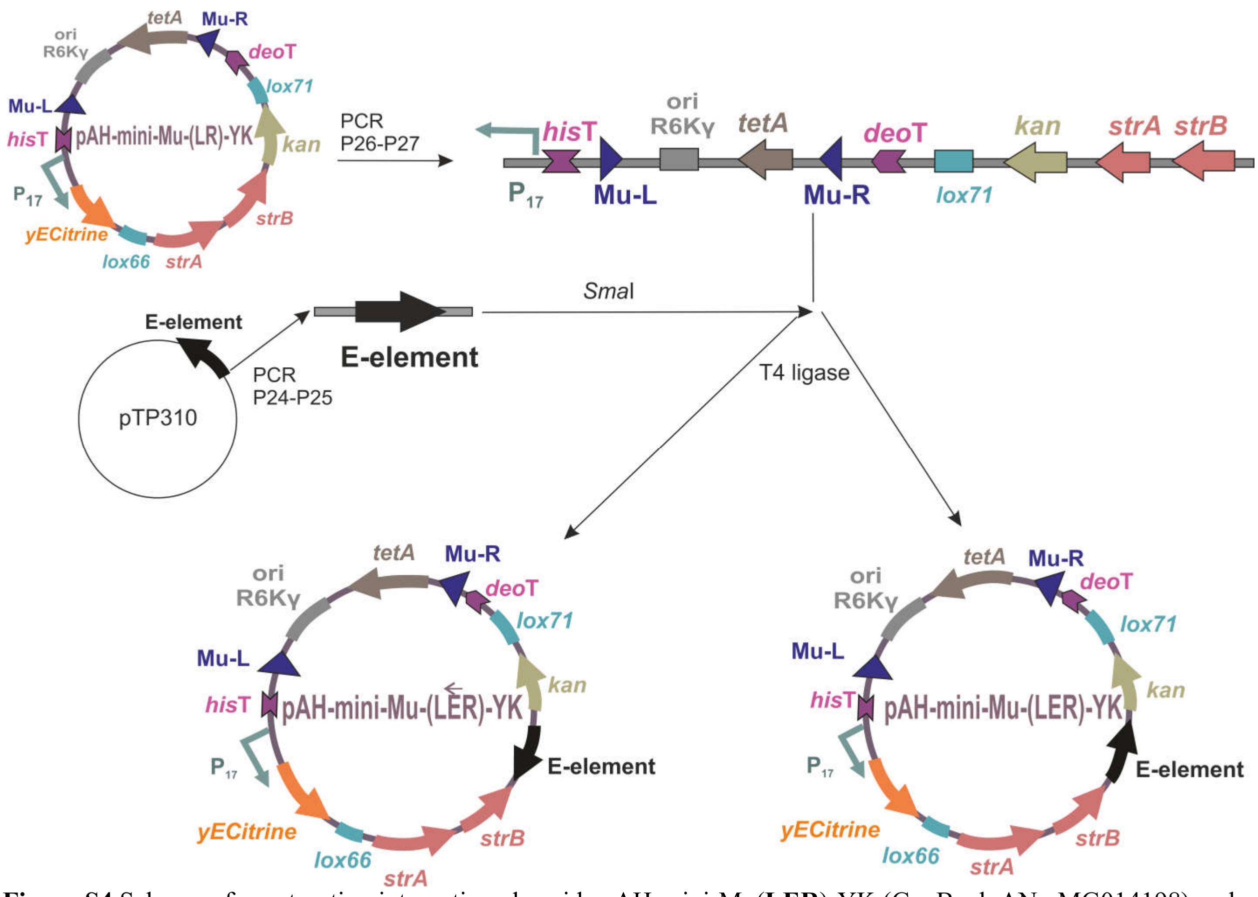

Figure S4 Scheme of construction integrative plasmids pAH-mini-Mu(**LER**)-YK (GenBank ANo MG014198) and pAH-mini-Mu(**L$\bar{E}$R**)-YK

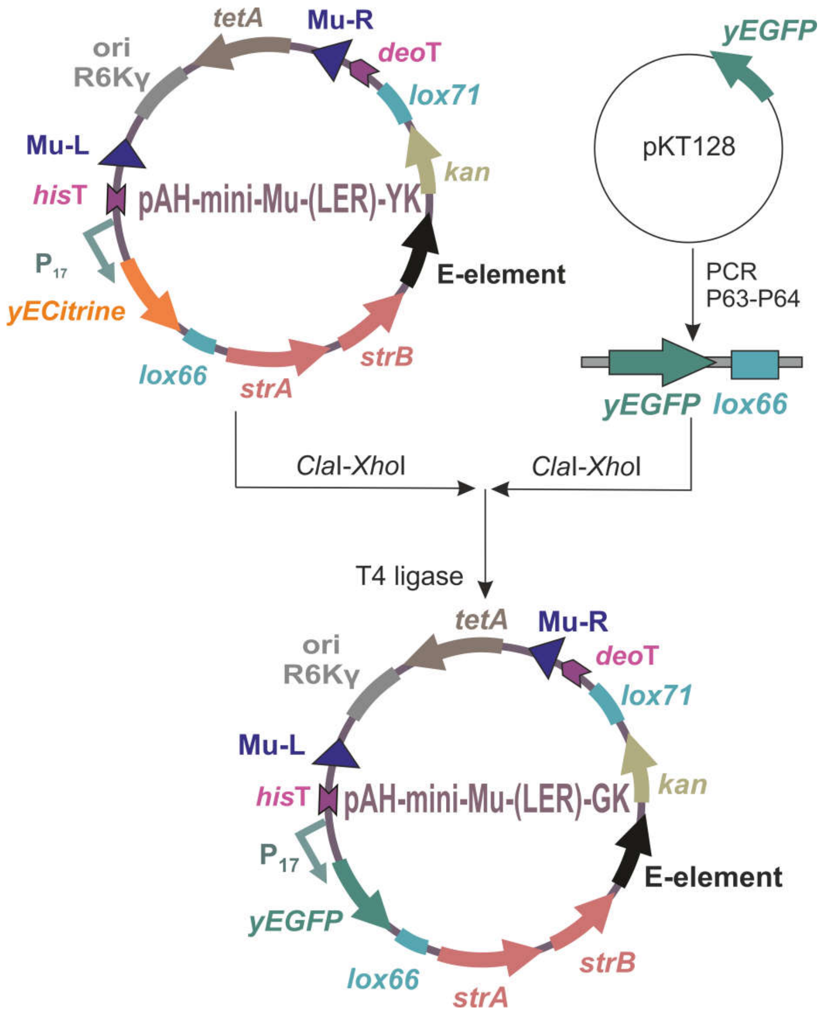

Figure S5 Scheme of construction integrative plasmid pAH-mini-Mu(**LER**)-GK

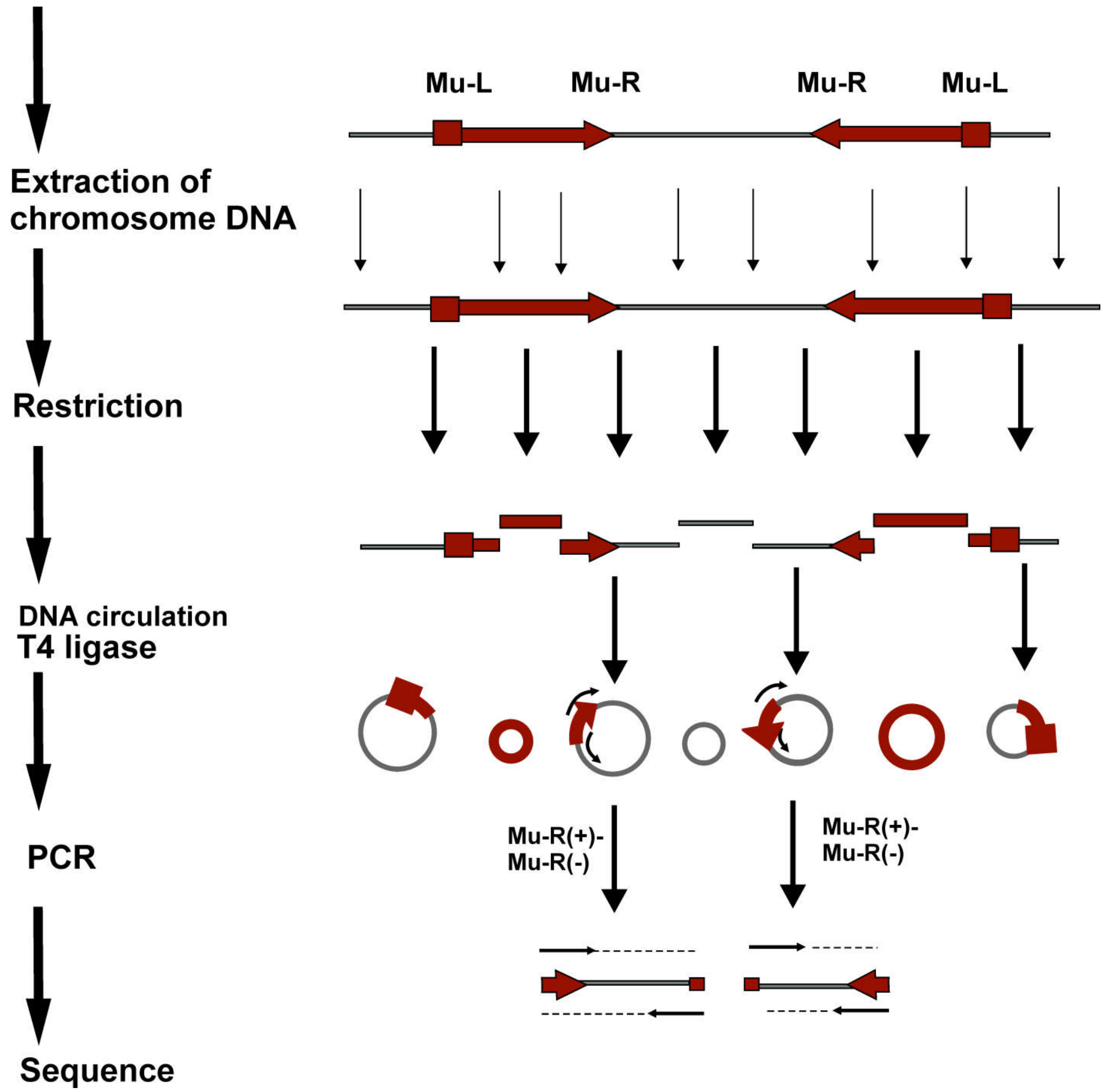

Figure S6 Scheme of confirmation of mini-Mu integration points

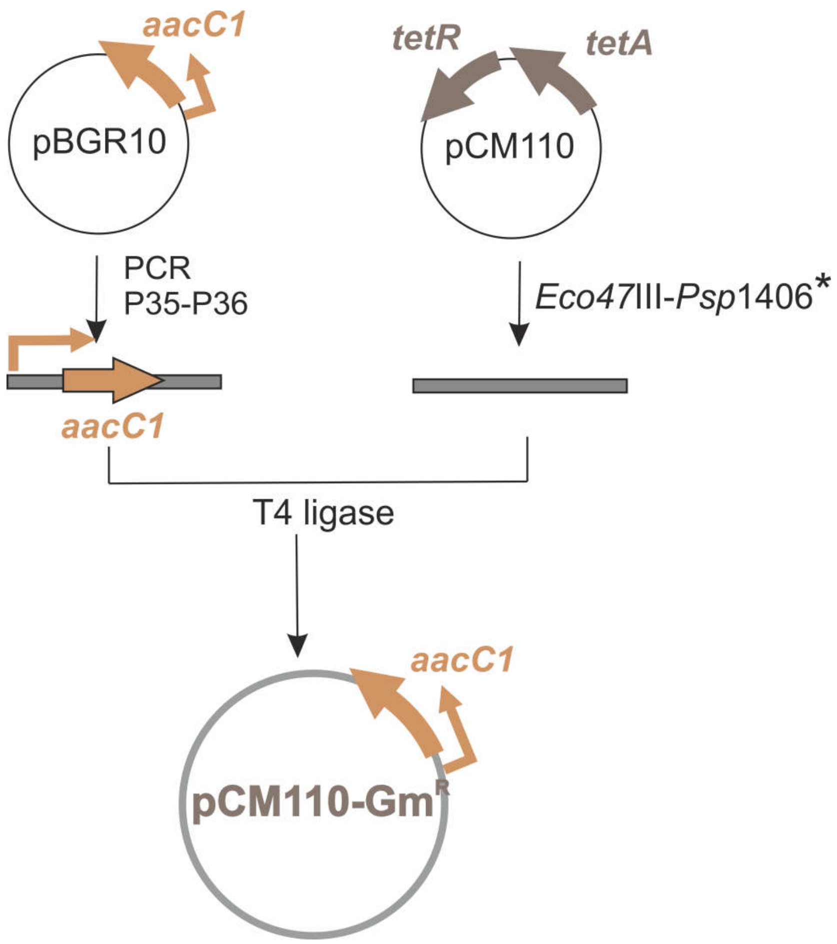

Figure S7 Scheme of construction pCM110-Gm^{R} plasmid

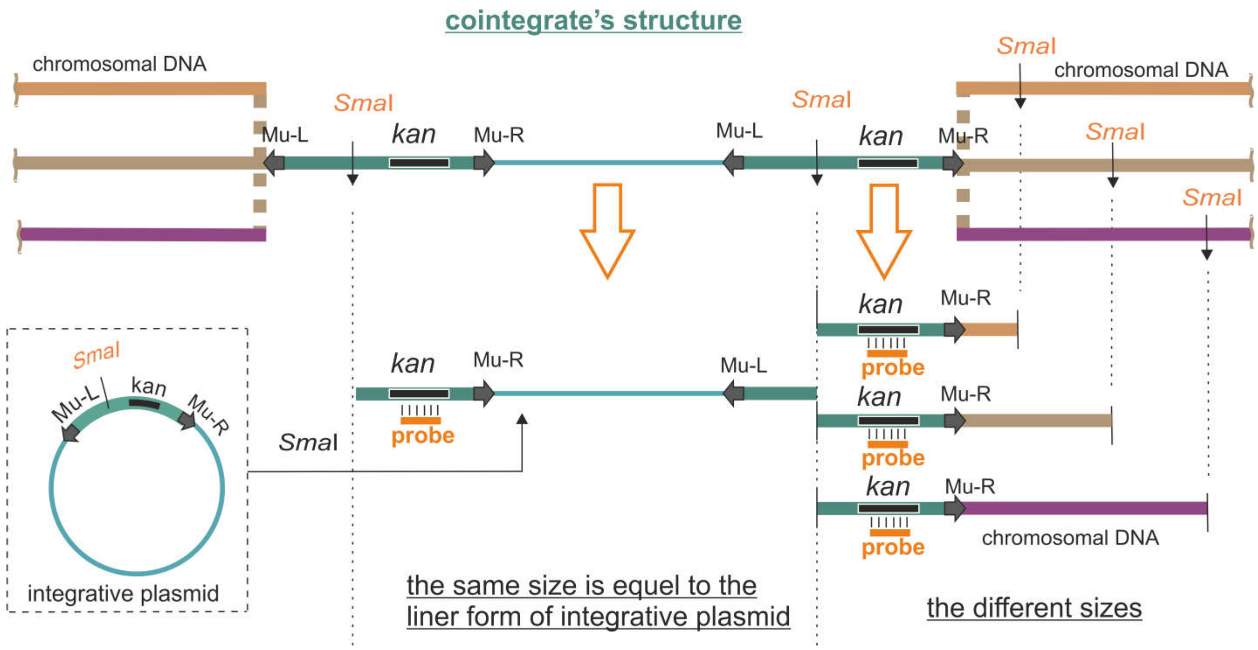

Figure S8 Explanation of **Figure 3B** – Scheme of the cointegrate structure formation and its resolution during replicative transposition of mini-Mu(**LER**)-YK in *C.glutamicum* chromosome followed by determination the number of mini-Mu(**LER**)-YK integration copies by Southern blot analysis

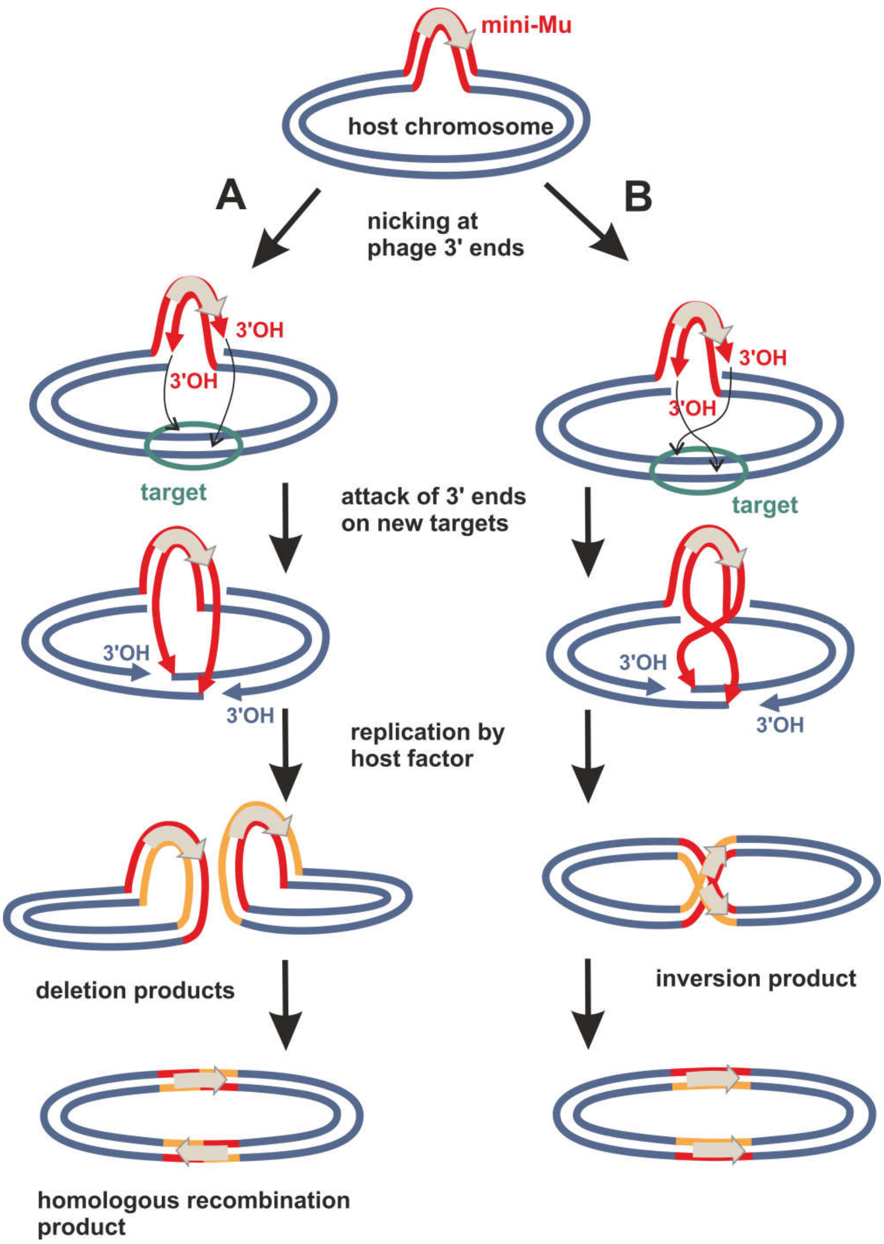

Figure S9 During lytic development of Mu bacteriophage intramolecular transposition takes place, within the same replicon, which involves many replicative events. Catalytically active MuA subunits of transpososome produce a nick at each 3' Mu end followed by transfer of resulting 3'-OH ends to target site, located on the same molecular. This reaction is assisted by helper-protein MuB. During this process the free 3'-OH groups make a 5-bp staggered cut of phosphodiester bonds on the complementary DNA strands in this target site. As result strand exchange product so-called "Shapiro intermediate" is formed, this resolved further by mechanism of replication. Besides the newly formed free 3'-OH ends of the intermediate product become the initiation sites –primers for Mu DNA replication. Depending on the choice of target strand- the same (**A**) or its complementary (**B**) the resolution leads to deletions (very rarely the lost of vital genes results to the deletion products fusion by homologous recombination) or inversions of the chromosome parts of the host genome

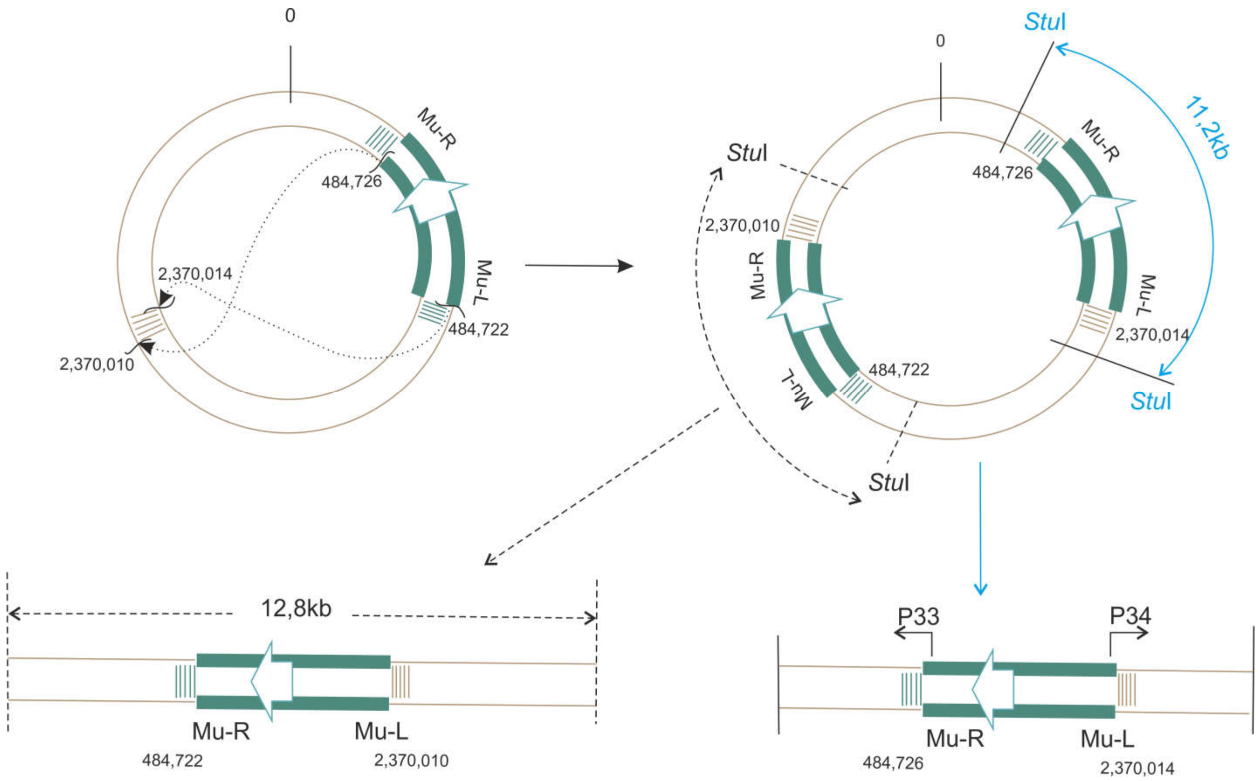

Figure S10 The scheme of the integration of two mini-Mu(**LER**)-YK units in the chromosome of the clone No10 produced by the intramolecular Mu-driven replicative transposition of the initially integrated one mini-Mu(**LER**)-YK unit
